# Effects and correctability of pile-up distortion using established figures of merit in time-domain diffuse optics at extreme photon rates

**DOI:** 10.1038/s41598-022-09385-5

**Published:** 2022-03-30

**Authors:** Elisabetta Avanzi, Anurag Behera, Davide Contini, Lorenzo Spinelli, Alberto Dalla Mora, Laura Di Sieno

**Affiliations:** 1 Dipartimento di Fisica, Politecnico di Milano, Piazza Leonardo da Vinci 32, 20133 Milan, Italy; 2 Istituto di Fotonica e Nanotecnologie, Consiglio Nazionale delle Ricerche, Piazza Leonardo da Vinci 32, 20133 Milan, Italy

**Keywords:** Near-infrared spectroscopy, Near-infrared spectroscopy, Characterization and analytical techniques

## Abstract

Time-domain diffuse optics (TD-DO) allows one to probe diffusive media with recognized advantages over other working domains but suffers from a poor signal-to-noise ratio (SNR) resulting from the need to build-up the histogram of single-photon arrival times with maximum count rates (CR) of few percent of the laser pulse rate to avoid the so-called “pile-up” distortion. Here we explore the feasibility of TD-DO under severe pile-up conditions with a systematic in-silico/experimental study evaluating the effects and correctability of the distortion by means of shared figures of merit. In-silico, we demonstrate that pile-up correction allows one the retrieval of homogeneous optical properties with average error < 1% up to a CR > 99%, while the optimal CR needed to detect localized perturbation was found to be 83%. Experiments reported here confirm these findings despite exhibiting higher accuracy errors in the retrieval of homogeneous optical properties and higher noise in the detection of localized absorption perturbations, but in line with the state-of-the-art systems. This work validates a new working regime for TD-DO, demonstrating an increase of the SNR at constant acquisition time, but also potentially leading in the future to previously unrealizable measurements of dynamic phenomena or in spatial scanning applications.

## Introduction

The use of light for materials analysis is spreading across the scientific community. In particular, diffuse optics (DO) exploits light scattering in diffusive media (e.g., biological tissues) to non-invasively estimate their chemical composition and microstructure by retrieving the absorption (${\mu }_{a}$) and reduced scattering (${\mu }_{s}^{^{\prime}}$) coefficients spectra of the sample in the visible or near-infrared range^1,2^. This finds application in the medical field (e.g., cancer diagnosis, brain functional imaging, etc.), as well as in other materials science branches (e.g., food, wood, pharmaceuticals analysis)^3^. The interest in DO is rapidly growing^4^, also paying attention to the definition and adoption of standardized performance assessment procedures for components, instruments, and measurement techniques^5–7^.

DO investigations are possible under different approaches. In particular, Time-Domain (TD) DO relies on the acquisition of the temporal profile of light exiting the probed sample to obtain its optical properties. Typically, TD-DO measurements are performed shining the medium with sub-nanosecond light pulses and collecting the re-emitted backscattered photons using time-resolved single-photon detectors and time-correlated single-photon counting (TCSPC) acquisition systems^8–10^ to reconstruct their distribution of times of flight (DTOF). The TD approach features a high information content as it allows one to independently retrieve ${\mu }_{a}$ and ${\mu }_{s}^{^{\prime}}$ even with a single measurement point^2,8^. When a reflectance geometry^2^ is employed, it also allows one to separate information coming from different depths in the medium since early arriving photons bring information from superficial regions, while late arriving ones can reach deep regions^7^. However, TD-DO measurements suffer from a limited signal-to-noise ratio^11^ (SNR) due to the need to reconstruct the DTOF one photon at a time, through periodic illumination of the sample with light pulses. In particular, TCSPC acquisitions ensure reliable DTOF reconstructions only if the ratio between the photon counting rate and the laser pulse rate is kept low (typically < 1–5%)^10^, thus avoiding the so-called “pile-up” distortion. When distortions are particularly concerning, the rate is further reduced even down to 0.1%^9^. In a pure Poisson process at constant rate, the SNR increases with the square root of the acquisition time^12^, therefore, long acquisition times are required to obtain a sufficient photon statistic. However, when measurements have to be fast either due to the need for following dynamic phenomena (e.g., functional brain imaging) or for spatial scanning requirements (e.g., optical mammography), the measurement has to cope with a low SNR.

Specifically, a common rule of thumb in the fluorescence decay analysis field suggests that keeping the count-rate (CR) below 5% allows one to maintain distortions in the order of 1%^10,13^. However, this rule is strongly linked with the lifetime analysis method adopted, since different parts of the DTOF are differently affected by pile-up distortions. For instance, in the measurement of homogeneous optical properties, TD-DO analysis also makes use of the rising edge of the DTOF, which is less affected by distortion with respect to the late region. To the best of our knowledge, there is no study in the field of TD-DO on the effect of pile-up distortion on the estimation of optical properties in diffusive media. Moreover, different pile-up correction methods have been proposed, but they all focus on applications other than DO, like lifetime measurements^13–18^ or laser ranging^19–21^. Yet, in TD-DO the pile-up limit is becoming a real bottleneck since the hardware developments are progressively increasing the intensity of the detected signal^8^ thanks to the availability of high-power lasers (tens of mW)^22,23^, large sensitive-area solid-state single-photon detectors (some mm^2^)^22,24^ and high-throughput TCSPC chains (from tens of millions to billions of counts per second -cps-)^22,25–27^. The pile-up effect can be mitigated: (i) by attenuating the detected signal, thus frustrating the potential gain produced by a large photon-collection area; (ii) by employing a large source-detector separation (ρ) to introduce a loss in the number of early photons, but this will also produce a loss of late ones^28^; (iii) by adopting a time-gated detection scheme^29^ to avoid detecting early-arriving photons, thus also introducing limitations in terms of unconventional noise sources^30^, non time-invariant effects^31^ and, typically, small detection area size^32^. Alternatively, parallelized architectures can limit the issue, combining different detectors and/or different independent TCSPC channels^26,33^, but this increases the system cost and complexity.

Few TD-DO studies have already explored the possibility to work under severe pile-up conditions. In a work by some of us^22^ it was demonstrated that TD-DO data could be acquired with reasonable accuracy and increased SNR by running at count rates (CR) of up to 76% of the laser pulse rate, by correcting the pile-up distortion with traditional methods^14^. Another work made use of pile-up distortion produced by a high CR to increase the number of early photons, so as to increase the spatial resolution of diffuse optical tomography acquisitions in transmittance geometry^34^. However, this latter approach is not feasible in reflectance geometry since it only allows probing the most superficial regions of the medium. Furthermore, transmittance geometry is often not feasible due to the thickness of the samples under analysis (e.g., the human head).

In this paper, we aim to validate a completely new operative regime for TD-DO, working well above the single-photon statistics. More in detail, we want to demonstrate the possibility to work in this regime in two paradigmatic situations for TD-DO applications: (i) the retrieval of optical properties of homogeneous media; (ii) the capability to detect an optical inhomogeneity buried in depth into a homogenous medium. To comprehensively study the proposed new operating regime, we consider situations starting from a nearly pile-up free case to severe pile-up conditions. To objectively assess the results, we make use of relevant DO figures of merit (FOMs) defined in existing and widely adopted performance assessment protocols for the investigation of both homogeneous and heterogeneous media (MEDPHOT and NEUROPT protocols^6,7^). Moreover, we verify the simulation results with experimental measurements. This work has two main limitations. First, it considers only a simple common pile-up correction algorithm^14^, leaving the implementation of more advanced correction techniques to future studies. Second, only classical pile-up is considered, neglecting second order effects that are present in some TCSPC detection chains, in particular when the detector dead time is lower than the TCSPC electronics one^13,17^.

## Results

### MEDPHOT protocol

**Figure 1:**
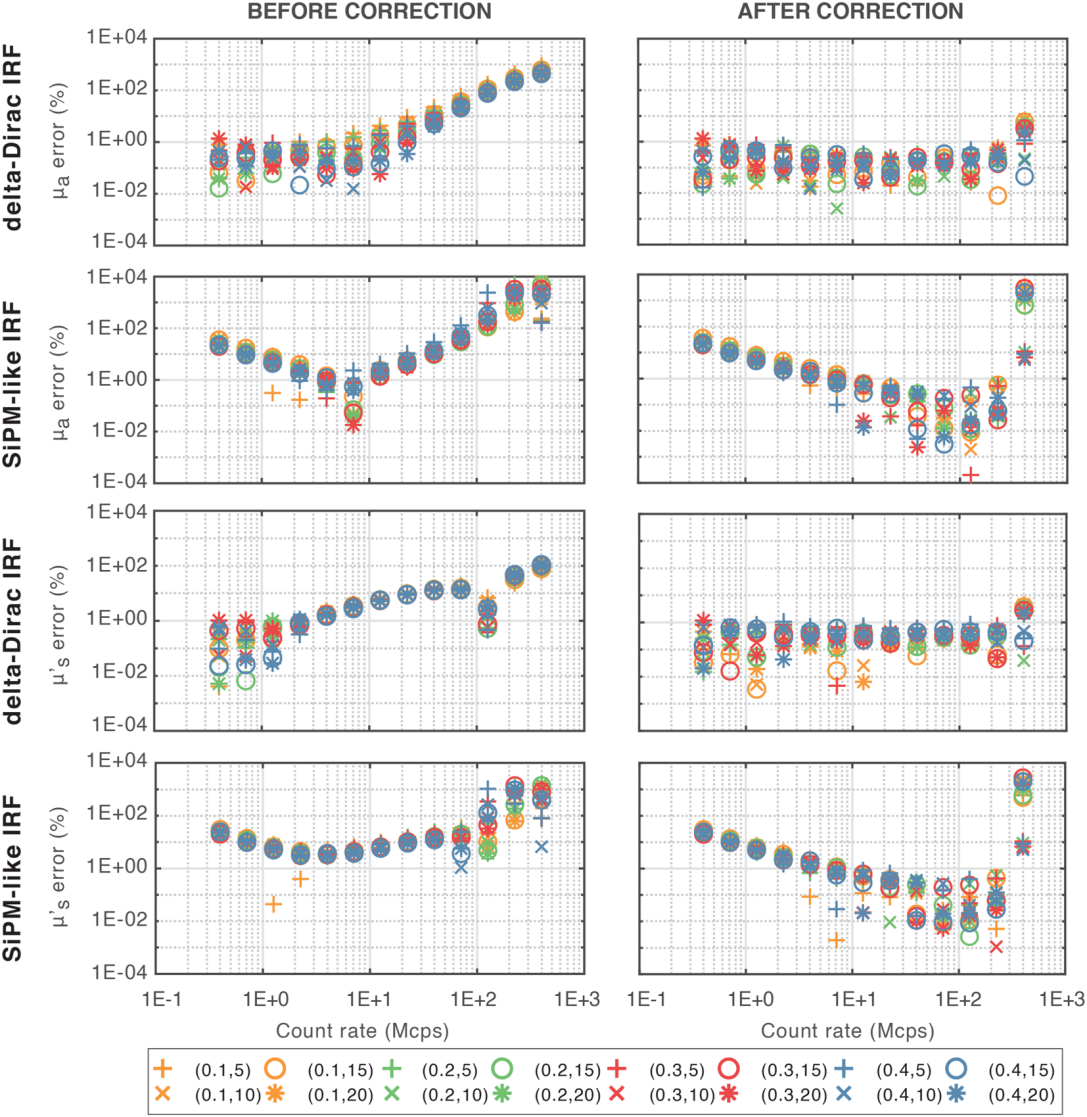
MEDPHOT protocol results for simulated data. Values obtained with delta-Dirac IRF are represented in odd rows, while those obtained with a SiPM-like IRF are in even ones. Each single point represents the absolute error for a particular phantom. Marker colors refer to different absorption coefficients, while marker types to different scattering ones. Absorption (first and second rows) and reduced scattering (third and fourth rows) coefficients errors are represented at various CRs, for all the phantoms. In all cases, both results before (left plots) and after (right plots) correction are shown.

Figure 1 shows the simulation accuracy results (absolute errors with respect to true values^6^) in recovering homogeneous ${\mu }_{a}$ and ${\mu }_{s}^{\mathrm{^{\prime}}}$ for each phantom at various CRs using both the before- and after-correction DTOFs. The odd and even rows show the results for ideal (i.e., delta-Dirac Instrument Response Function -IRF-) and realistic (i.e., SiPM-like IRF) systems, respectively. It is worth noting that the CRs shown in all plots refer to the situation after pile-up correction (for the equivalence in terms of saturated count-rate and its percentage with respect to the repetition rate of the laser, see the first 3 columns of Table 1). Tables with values of average errors in the retrieval of absorption and reduced scattering coefficients can be found in Supplementary Materials (Tables S1, S2, for delta and SiPM-like IRF respectively). Looking at the results obtained before correction for the ideal system, we can see average errors of less than 1% at CR ≤ 7.1 Mcps for ${\mu }_{a}$ and CR ≤ 2.3 Mcps for ${\mu }_{s}^{^{\prime}}$. This is expected since the system is working within (or close to) single-photon statistics. At larger CRs, due to pile-up, the error on optical property retrieval starts diverging for both optical coefficients, reaching a maximum value of 563.44% for ${\mu }_{a}$ and 93.04% for ${\mu }_{s}^{^{\prime}}$ in the worst case (i.e., at CR = 400.0 Mcps). Instead, when looking at the data after correction the average errors are smaller than 1% up to CR = 224.9 Mcps for both coefficients. Even in the extreme case (i.e., 400.0 Mcps), the average errors are limited (2.84% for ${\mu }_{a}$, 2.13% for ${\mu }_{s}^{^{\prime}}$). Considering now the simulations of the realistic system, results before correction exhibit errors with a descending trend up to CR = 7.1 Mcps for ${\mu }_{a}$ and CR = 2.3 Mcps for ${\mu }_{s}^{^{\prime}}$. However, ${\mu }_{s}^{^{\prime}}$ average error is greater than 1% at all CRs, while ${\mu }_{a}$ average error is less than 1% only at 4.0 and 7.1 Mcps. At higher CRs, similarly to the delta-Dirac case, errors rise resulting in not only an increase in mean value, but also in a significant dispersion across phantoms. Looking at the results after correction, we assist to a similar descending trend of the error, but, thanks to the correction, average errors of less than 1% may be obtained over a CR range of 7.1–224.9 Mcps for both optical coefficients. Generally speaking, the initial decreasing trend in the errors can be ascribed to the complex interplay between instrument response shape, background noise and Poisson statistics. Indeed, an increasing CR allows one to boost the measurement dynamic range with respect to the background noise and to reduce the relative effect of Poisson noise along the DTOF. Such phenomenon can be barely noticed also in the delta-Dirac case, where however the absence of background noise and the ideal response shape reduce the slope of this trend.

**Table 1:** CRs used for simulations before (CR_sat_) and after (CR) correction (first and third columns).

CR_sat_ [Mcps]	CR_sat_ [% Exc. rate]	CR [Mcps]	CR [% Exc. rate]
0.40	1.00	0.40	1.00
0.71	1.76	0.71	1.78
1.25	3.11	1.26	3.16
2.19	5.47	2.25	5.62
3.81	9.52	4.00	10.00
6.52	16.29	7.11	17.78
10.84	27.11	12.65	31.62
17.21	43.01	22.50	56.23
25.29	63.21	40.00	100.00
33.24	83.11	71.13	177.83
38.31	95.77	126.50	316.23
39.86	99.64	224.94	562.34
40.00	100.00	400.00	1000.00

The percentage of the excitation rate is reported (second and fourth columns).

**Figure 2:**
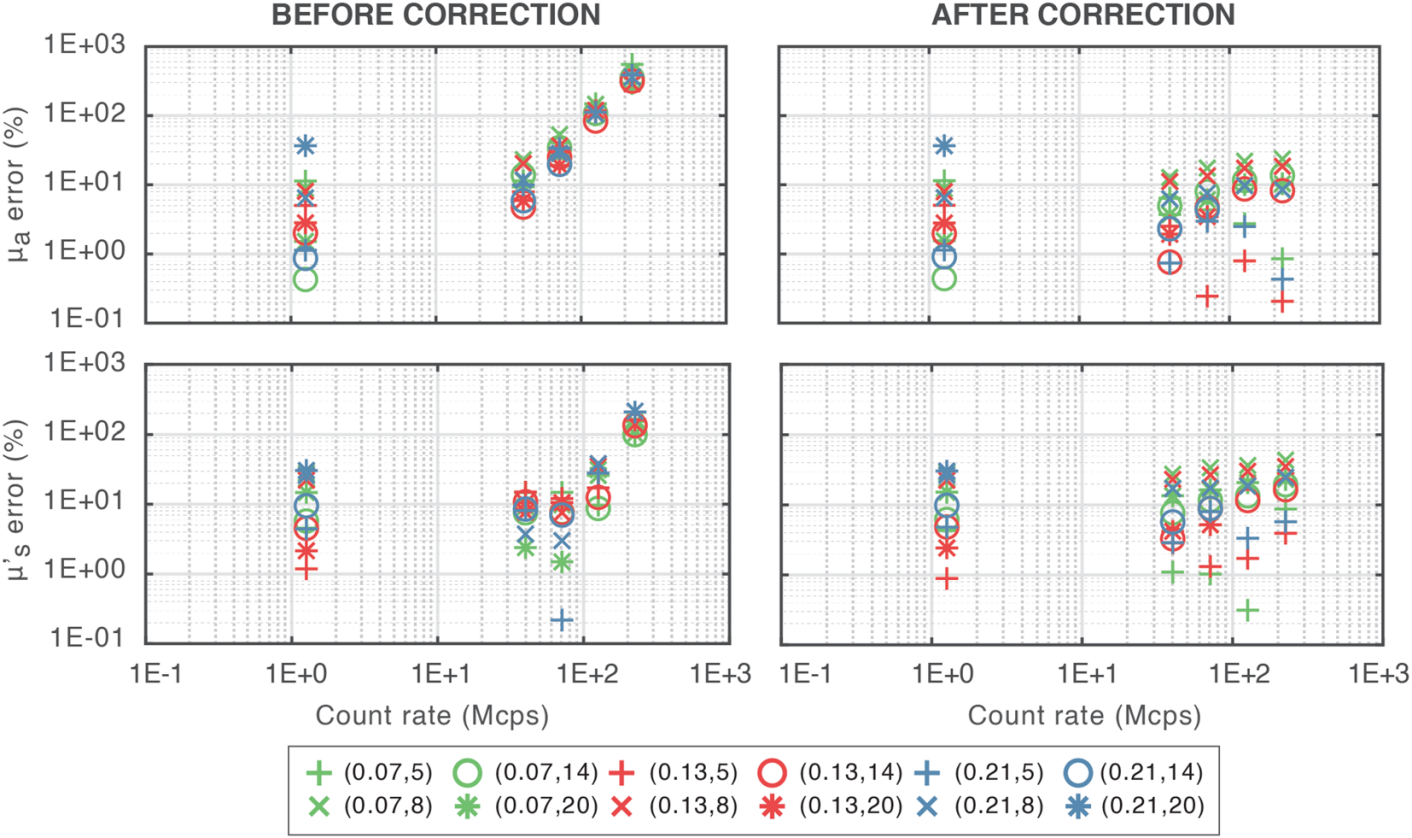
MEDPHOT protocol results for experimental data. Each single point represents the absolute error for a particular phantom. Marker colors refer to different absorption coefficients, while marker types to different scattering ones. Absorption (first row) and reduced scattering coefficients (second row) errors are represented at various CRs, for all the phantoms. In all cases, both results before (left plots) and after (right plots) correction are shown.

Figure 2 depicts, for each phantom, the accuracy results (absolute errors with respect to conventionally true values^6^) in recovering homogeneous ${\mu }_{a}$ and ${\mu }_{s}^{^{\prime}}$ at varying CRs in the experimental case, both before and after applying pile-up correction.

Despite showing on average higher errors with respect to simulation results (as expected and in line with those obtained by using state-of-the-art instruments^35^), the effectiveness of the pile-up correction is confirmed as the ${\mu }_{a}$ average error is reduced below 10% at all the investigated CRs. On the other hand, ${\mu }_{s}^{^{\prime}}$ has mean errors smaller than 10% only for 1.2 and 40.0 Mcps, while errors smaller than 20% are obtained up to 224.9 Mcps. However, this still shows the goodness of the pile-up correction, allowing to reduce the average ${\mu }_{s}^{^{\prime}}$ error from about 120% to 17.4% at the extreme CR of 224.9 Mcps. It is worth noting that, generally speaking, some differences between accuracy errors in the retrieval of ${\mu }_{a}$ and ${\mu }_{s}^{^{\prime}}$ are expected since their information is mainly encoded into two different regions of the DTOF (i.e., in its decay tail slope and peak time and shape, respectively). Also in this case, the average error values are reported in Table S3 in the Supplementary Materials.

### nEUROPt protocol

**Figure 3:**
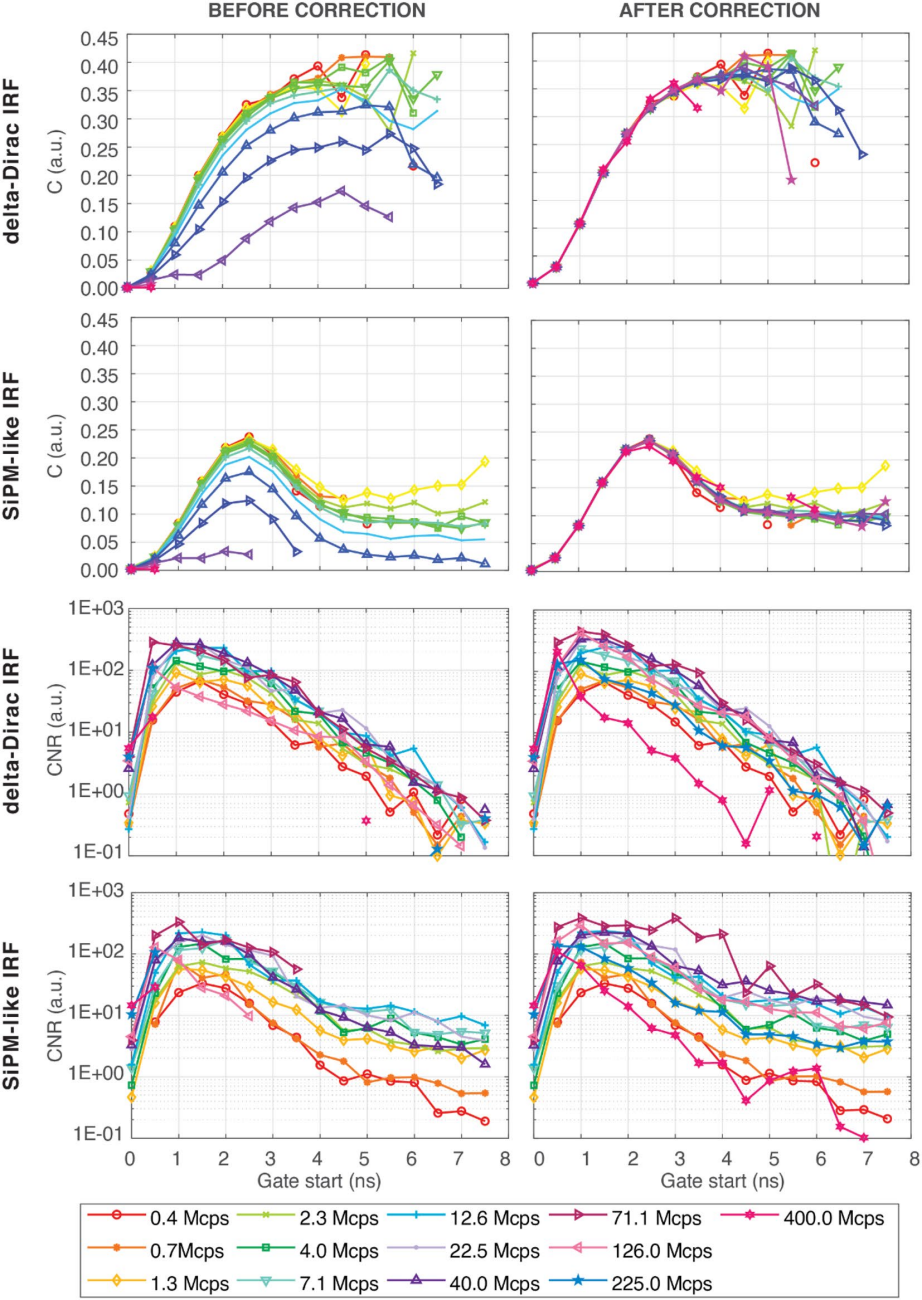
nEUROPt protocol results for simulated data. Values obtained with delta-Dirac IRF are in odd rows, while those obtained with a SiPM-like IRF are in even rows. Contrast (first and second rows) and CNR (third and fourth rows) values are shown at subsequent gates (evaluated from the IRF peak) for different CRs (colours and markertypes). In all cases, both results before (left plots) and after (right plots) pile-up correction are shown.

Figure 3 reports the simulation results about the FOMs defined in the nEUROPt protocol^7,36^, i.e., contrast (C—relative difference in the number of photons caused by the absorption change, with respect to the homogenous state, due to the presence of the perturbation) and contrast-to-noise ratio (CNR—index that evaluates the strength of the contrast with respect to noise fluctuations) *vs*. the starting time of subsequent 500-ps width gates (evaluated from the IRF peak) before and after correction, considering both the ideal (odd rows) and the realistic systems (even rows). Tables with values of C and CNR within each gate and at varying CRs can be found in Supplementary Materials (Tables S4 and S5 for delta IRF and Tables S6 and S7 for SiPM-like IRF).

Looking at the data obtained before correction for the ideal system, we can observe that generally the computed C decreases with the CR. This phenomenon is expected as the CR is set at the targeted value for the unperturbed case (i.e., without perturbation in the medium), thus resulting lower in the perturbed case (i.e., with perturbation in place that absorbs a fraction of photons). Therefore, the pile-up distortion has typically more effect on the unperturbed DTOF with respect to the perturbed one, thus diminishing the difference in the number of detected photons between the two cases. Furthermore, the C is not always visible in each gate. This is related with the minimum visibility condition used (i.e., CNR ≥ 1 within the gate), and it is especially true for the last two CRs, where the contrast is barely noticeable up to 0.5–1.0 ns. In terms of CNR, we instead see its constant enhancement with CR up to 40.0 Mcps (for few gates up to 71.1 Mcps) even if some isolated points are larger than 0 for later gates but still lower than 1 (their presence is related with noise fluctuations). On the other hand, after pile-up correction, C is restored to values defined by single-photon statistics in the majority of cases (some late points are still missing due to the condition of CNR < 1). The effect of the pile-up correction is especially visible in the CNR for CR ≥ 71.1 Mcps. Indeed, we can see average improvements of at least 46% between before and after applying the pile-up correction (e.g., for 224.9 Mcps the CNR after correction is, on average, even 224% larger than before correction case). Furthermore, even if the optimum CNR is at 71.1 Mcps, it is worth noting that CNR at 126.5 Mcps and 224.9 Mcps is higher than at 0.4 Mcps (within single-photon statistics) for most of the gates. As a result, the CNR post-correction is better than CNR obtained within single-photon statistics. The NaN values present in Table S5 in the Supplementary Materials are related to the absence of signal due to the high pile-up distortion in such late channels. Thus, when the CNR is computed, both numerator and denominator are null, thus producing a NaN output.

When considering the results obtained for the realistic system, it is possible to notice a different trend in the C function. In particular, while a delta-Dirac response produces C functions which continuously increase with the gate delay generally up to 4.5–5.0 ns (as expected^28^), the finite IRF shape of the realistic system affects the late gates, resulting into a reduction of the C due to the SiPM response tail^37^. However, similarly to the delta-Dirac case, without pile-up correction the C reduces at high CR. For CR ≥ 71.1 Mcps the points associated to gates after the peak start to be eliminated because the CNR is < 1 (extreme condition is again with 224.9 and 400.0 Mcps where only two points are represented). The CNR before correction is characterized by a steady increase in absolute values up to about 71.1 Mcps, when it reaches a maximum condition. This last CR, however, is characterized by the truncation of the significant gates at roughly 3.0–3.5 ns, after which negative values of CNR are present. When pile-up correction is applied, the values of C are restored to what expected under single-photon statistics. It has to be noticed that at 400.0 Mcps, the C in some late gates (from 4.5 to 5.0 ns) is not visible since the CNR < 1, leading to an insufficient significancy of the C with respect to the noise fluctuations. The pile-up correction also improves the CNR as for instance, at 71.1 Mcps, the CNR is now larger than 1 at all delays up to 7.5 ns. However, the CNR obtained at 126.5, 224.9 and 400.0 Mcps, except for the first gate (0–0.5 ns), is lower when compared to CR leading to maximum CNR (71.1 Mcps). Still, at 126.5 and 224.9 Mcps the CNR after correction is typically better than at low CRs.

**Figure 4:**
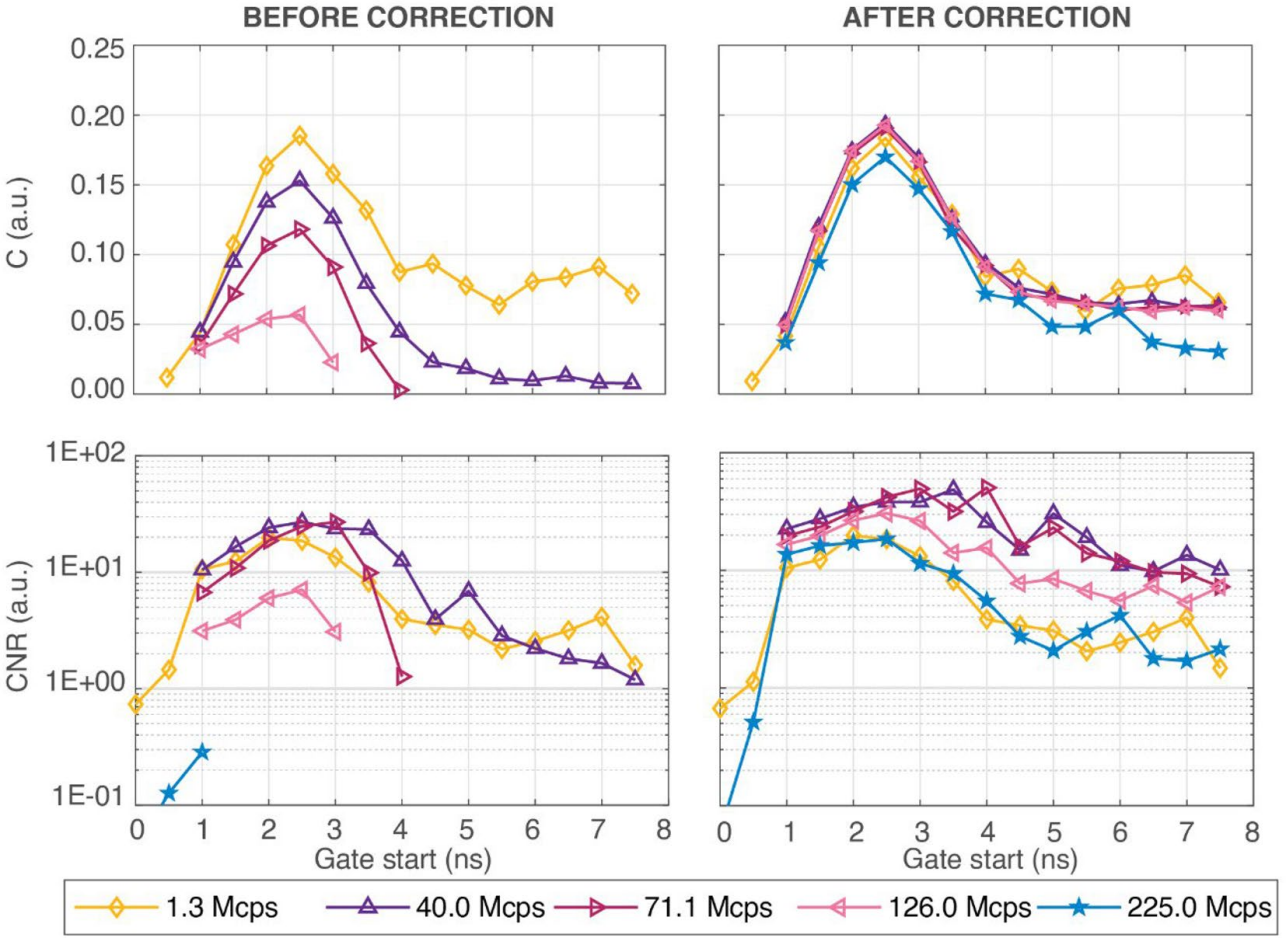
nEUROPt protocol results for experimental data. Contrast (first row) and CNR (second row) values are shown at subsequent gates (evaluated from the IRF peak) for different CRs (colours and marker types). For both FOMs, results before (left plots) and after (right plots) pile-up correction are shown.

Figure 4 shows C and CNR values resulting from experimental data at various gate delays for different CRs, utilizing both before- and after-correction DTOFs. Tables S8 and S9 in Supplementary Materials show C and CNR computed for measurements. When examining the plots before pile-up correction, it is clear the deterioration of the C as the CR grows. Furthermore, all the C data at 224.9 Mcps are not available due to the visibility condition unfulfillment (CNR < 1). At excessive CR, pile-up distortion has a major impact on the CNR as well. Indeed at 71.1 and 126.5 Mcps, late gates (from 4.5 and 3.5 ns, respectively) are absent, while for 224.9 the condition CNR ≥ 1 is not satisfied for all the considered gates. After correction, the C is mostly recovered to single-photon statistic levels, and the CNR improves as the CR grows up to 71.1 Mcps, after which the pile-up correction starts becoming less effective. In any case, after correction, the results always have C > 1% and CNR ≥ 1 at delays ≥1 ns; hence, the visibility of the perturbation is always fulfilled even when working well beyond typical operating conditions.

## Discussion

When correction algorithm is used, our research reveals that for TD-DO systems working under severe pile-up conditions is possible. Indeed, simulations show that recovering optical characteristics of homogeneous media may be done with high accuracy for CR well beyond single-photon statistics (errors smaller than 1% are present up to 224.9 Mcps -corresponding to 99.64% of the laser pulse rate- for the ideal system and between 7.1 and 224.9 Mcps -corresponding to a range between 16.29 and 99.64% of the laser pulse rate- for the realistic one), provided that a suitable correction algorithm is applied. Furthermore, despite at low CRs the ideal system outperforms the realistic one (as expected due to the interplay between instrument response shape, background noise and Poisson statistics), except for the 400.0 Mcps case, when the CR grows, the realistic system achieves an average error comparable (i.e., same order of magnitude) with the ideal one. We may find an optimal condition at 71.1 Mcps for both ${\mu }_{a}$ and ${\mu }_{s}^{^{\prime}}$, with an average error of less than 1% and a minimum dispersion across different optical characteristics.

It is worth noting that the accuracy errors and linearity FOMs simulated in this work are much better if compared with the majority of TD-DO systems, where for instance accuracy errors of few tens percent can be found^6^. This is partially due to the fact that, for the MEDPHOT protocol study, we use the same model both to produce DTOFs and to fit them to retrieve the optical coefficients used to compute the errors. This “inverse crime” (as referred in the TD-DO field on the use of the same procedure for data production and analysis) is in part desired to highlight just the effect of pure pile-up, without potential crosstalk with the validity of the diffusion approximation in case of optical properties combining high absorption with low scattering. In parallel, the use of phantoms with low absorption in the experimental part of this study, despite motivated by the need for high photon detection rates, contributed to limit this effect. However, the investigation of the combination of this effect with pure pile-up, if desired, is quite straightforward and could be easily implemented by using a Monte Carlo algorithm for forward simulations^38,39^, while maintaining the procedure used in this study for data analysis.

The experimental tests confirm the feasibility of the proposed approach. Indeed, absolute errors are always < 7% for absorption coefficient and always < 18% for reduced scattering coefficient, with weak CR effects in terms of dispersion of the error among different phantoms. Despite the high CR, these errors are in line with those of state-of-the-art spectroscopy systems^35^. Most probably because of the higher average errors in the experimental case, differently from simulations, the after-correction MEDPHOT results do not show a decreasing error trend with increasing CR. However, the best measured accuracy values are achieved at CR = 40.0 Mcps, since they have the lowest average error (i.e., − 0.89% for ${\mu }_{a}$ and 8.10% for ${\mu }_{s}^{^{\prime}}$).

Measurement data are not directly comparable to simulations since: (i) the optical parameters employed for simulations do not exactly match those of the MEDPHOT phantoms, and (ii) the experimental set of optical properties considers phantoms with an absorption coefficient which is at the maximum about half of the simulated one (indeed, due to the reduced signal reaching the detector, it was not possible to work up to a condition of severe pile-up with highly absorbing samples).

The effect of the correction is visible also on linearity and crosstalk (average values and standard deviation among different optical properties, for both linearity and crosstalk slopes, are reported in Supplementary Materials Tables S1, S2 and S3). Indeed, for ideal system simulations, the linearity has almost a unitary slope for absorption and scattering up to 224.9 Mcps. The coupling of absorption over scattering is reduced as well, thus slopes close to 0 are obtainable up to 400.0 Mcps (with a sensitivity limited to the 5th digit). The correction acts on the crosstalk of ${\mu }_{s}^{^{\prime}}$ over ${\mu }_{a}$ up to 224.9 Mcps (where slopes values are closer to 0 if compared to the before-correction cases). Instead, at 400.0 Mcps the correction is insufficient (an average slope of about − 1.16 is obtained). Similar advantages on linearity and crosstalk are visible for the simulations of the realistic system. However, the correction is effective for CR < 400.0 Mcps. Indeed, at 400.0 Mcps the average slope for absorption and scattering linearity is 8.5 and 18.8, respectively, while the slope is about 0.24 absorption–scattering crosstalk and − 6.76 for the scattering-absorption one. Moreover, the experimental results confirm the correction advantages. Thus, linearity is close to unity for ${\mu }_{a}$, however for ${\mu }_{s}^{^{\prime}}$ there is a deviation from the ideal slope of up to 30%. Moreover, negligible ${\mu }_{a}$ over ${\mu }_{s}^{^{\prime}}$ crosstalk is present, after the application of the pile-up correction. While a strong coupling between measured ${\mu }_{s}^{^{\prime}}$ and conventionally true ${\mu }_{a}$ is visible, even if the correction is applied.

According to the nEUROPt simulations, for both ideal and realistic systems, up to a CR of 224.9 Mcps (corresponding to 99.64% of the laser pulse rate), the C is recovered thanks to the pile-up correction. Further, an increase in the CR up to 71.1 Mcps (corresponding to 83.11% of the laser pulse rate) leads to visibility of C at the latest delays (for the ideal system). Since the arrival time of photons encodes the depth reached during their propagation in the random medium, the visibility of C in the late gates allows for the detection of deeper perturbations. Thus, we can select CR = 71.1 Mcps as the ideal working point, where the C is recovered and visible up to a delay of 7.5 ns (ideal system) or 8.0 ns (realistic system) and the CNR is maximized if compared to results obtained at standard CRs. These results are obtained thanks to the enhancement of the signal with respect to the noise that affects the late part of the DTOFs, allowing, for instance, to overcome the CNR obtained at 1.26 Mcps. Despite being 71.1 Mcps the ideal working point, the use of a CR of 126.5 or 224.9 Mcps (corresponding to 95.77% or 99.64% of the laser pulse rate) is not detrimental, leading to a CNR that is at least equivalent to the measurement performed within single-photon statistics, without affecting the C.

In contrast to what was mentioned for the MEPDHOT protocol, measurements may be compared to simulations in the nEUROPt case. For both C and CNR, a comparable shape between measurements and simulations can be detected. However, in line with previous works^36^, the C obtained with the dynamic phantom in single-photon statistics conditions is slightly smaller than that acquired through simulations. As expected, this is also true for data acquired under pile-up conditions after correction, since C is restored to values achieved at low CRs. The C values after-correction acquired at 40.0, 71.1 and 126.5 Mcps are almost identical. Thus, thanks to late gates visibility and CNR maximization, these three extreme CRs may be suitable operating conditions, without the need to precisely tune the CR (e.g., without the need for precise signal equalization in the case of multichannel systems).

For the detection of localized perturbation, the high CR regime pointed out an enhanced sensitivity to deep layers. This is comparable to what has been accomplished in the field using time-gating, but here achieved with less sophisticated technology^23^.

The nEUROPt study represents a first basic step inside the vast field of heterogeneous structures. On one hand the results reported here may not be easily replicated when targeting more complex structures like multilayer samples or complex three-dimensional structures (usually approached with tomographic strategies). On the other hand, the promising results here reported could represent the basis for future works in this direction.

In general, we can observe that nEUROPt offers fewer advantages than MEDPHOT when working with serious pile-up distortion. This is due to the fact that pile-up causes a loss of late photons, which is then artificially estimated by the pile-up correction. In particular, this algorithm acts by magnifying not only the signal but also the noise, thereby causing a reduction with the CR of the CNR at extreme CR conditions.

As anticipated in the introduction, there are two major limitations in this study. First, we only explored the use of a simple correction algorithm that was proposed in the literature decades ago^14^, which however demonstrated here to be very effective when applied to TD-DO. This is a basic approach that does not rely on any hypothesis such as corrected curve knowledge or time bin homogeneity. Indeed, the correction connects the experimental observation to the true probability of detecting a photon at a given channel, assuming that additional photon detections might occur in one cycle. Future studies could evaluate whether or not the use of more advanced pile-up correction algorithms can produce better results. Second, our simulations just examined classical pile-up effects, neglecting other possible distortions caused by secondary dead time effects^13,17^ like, e.g., localized bumps occurring when the detection chain has a dead time shorter than the DTOF temporal range. These tests indeed require the use of more advanced hardware for experimental validation and more complicated data correction methods. However, the advantages of applying a simple pile-up correction to TD-DO data also affected by other dead-time effects have been already demonstrated experimentally^22^, reaching percentages of CR of about 95.8% (i.e., for a 40-MHz laser system equivalent to 127.3 Mcps after pile-up correction).

In conclusion, we demonstrated, using pile-up correction, the feasibility of TD-DO under extreme pile-up conditions, paving the way for a completely new operating regime. In-silico, we reported the possibility to retrieve homogeneous optical properties with an average error smaller than 1% up to a CR larger than 99% of the laser repetition rate, whereas the optimal CR for detecting localized perturbation was discovered to be around 83%. Experiments confirmed these findings. Indeed, despite the expected increased accuracy errors in the retrieval of homogeneous optical properties and the higher noise in the detection of localized absorption perturbations, the results obtained under extreme pile-up conditions are, after correction, better than (or at least in line with) those of state-of-the-art systems.

## Methods

### Simulations

Simulations are carried out using DTOFs obtained by solving the radiative transfer equation under the diffusion approximation (i.e., light propagation dominated by multiple scattering) for a semi-infinite medium, either homogenous or containing a localized absorbing perturbation^40^. Heterogenous DTOFs have been simulated using an 8th-order perturbative solution of the diffusion equation^41^.

**Figure 5:**
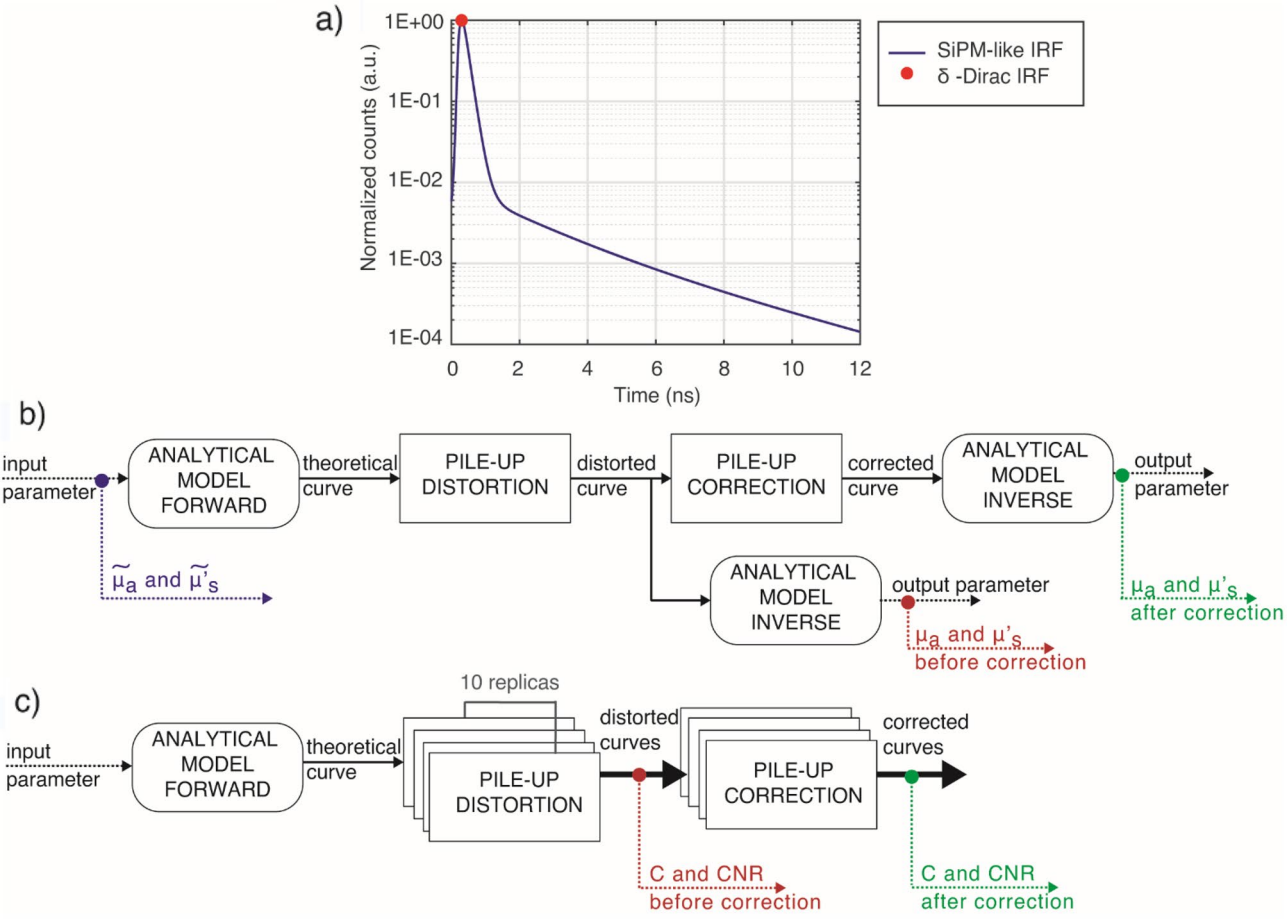
(**a**) Representation of IRFs used for simulations. Schematics of data generation and analysis for both MEDPHOT (**b**) and nEUROPt (**c**) data.

While the ideal system is modeled with a delta-Dirac IRF, for the SiPM-based system we convolve the theoretical reflectance waveforms with the IRF of a typical SiPM-based TD DO system^42^ considering, in this way, its finite duration and the presence of exponential tails. To avoid temporal shifts between the two systems, we aligned their IRF peaks as shown in Fig. 5a.

As simulation parameters we use: a laser rate of 40 MHz, a TCSPC bin width of 5 ps, a ρ of 3 cm and a diffusive medium with a refractive index of 1.55 (epoxy resin, i.e., the more common material of phantoms used in DO).

We consider 13 CRs (see Table 1) with values going from 0.01 to 10 times the laser rate; thus, CR values span both well-within and extremely-beyond single-photon statistics conditions (typically CR< 1–5 % laser rate). To convert the CR values before correction (CR_sat_) into the ones after correction (CR), it is possible to use the following equation:

1 $$CR_{sat} = f_{laser} *\left( {1 - \exp \left( -{\frac{CR}{{f_{laser} }}} \right)} \right)$$

where ${f}_{laser}$ is the laser rate. While CR is the potentially detectable photon rate, CR_sat_ is the photon rate processed by the timing board, which cannot overcome in our case ${f}_{laser}$.

We simulate DTOFs over a time scale of 12 ns and we introduce a Dark Count Rate (DCR) of 100 kcps to the SiPM-based system (no DCR is introduced for the ideal system). Furthermore, to have consistency between measurements and simulations, the SiPM data have been generated considering a detector with elliptical area (axis dimensions of 1 and 1.78 cm) and with major axis orthogonal to the ρ.

For the spectroscopy of homogeneous samples, we study a set of media characterized by 4 ${\mu }_{a}$ values (from 0.1 to 0.4 cm^−1^ at step of 0.1 cm^−1^) combined with 4 ${\mu }_{s}^{^{\prime}}$ values (from 5 to 20 cm^−1^ at step of 5 cm^−1^). For the detection of a localized perturbation, we consider a background medium with ${\mu }_{a}$ = 0.1 cm^−1^ and ${\mu }_{s}^{^{\prime}}$ = 10 cm^−1^, and a cubic absorbing perturbation with a volume of 1 cm^3^ and $\Delta {\mu }_{a}$  = 0.17 cm^−1^ placed at half of the ρ at two depths below the surface: 1.5 cm (heterogeneous case) and 10 cm (homogeneous case).

The classical pile-up distortion has been added to each DTOF using a Matlab code based on random Poisson launches. The algorithm treats each DTOF as a time-dependent detection probability distribution, adjusting its amplitude to obtain the desired CRs. This replicates what occurs when laser pulses excite a diffusive medium by also introducing Poisson noise. Each distorted DTOF is characterized by its own photonic noise, hence, for the detection of localized perturbation we simulate 10 replicas for each CR to also evaluate the standard deviation of counts (as required by nEUROPt protocol).

### Measurements

A high-power 670 nm laser system (LDH-P-C-670M driven by PDL 828 Sepia II, Picoquant GmbH, Germany) is operated at 4 MHz and directly coupled to an optical fiber (core diameter 600 μm, step index) that served as the input of a fiber-to-fiber u-bench, which included a variable optical attenuator for setting the correct photon counting rate. The output of each u-branch is another equivalent fiber, which is inserted in the probe that also host the detector at ρ = 3 cm. This is a home-made SiPM module^42^ based on 1.3 × 1.3 mm^2^ detector (S13360-1350PE, Hamamatsu Photonics K.K., Japan) with a DCR of 713 kcps. A black mask was placed in front of the SiPM module input glass window to limit the photon collecting area to an ellipse with axis dimension of 1 cm and 1.78 cm (major axis orthogonal to ρ). A TCSPC board (SPC 130, Becker and Hickl GmbH, Germany) is utilized as timing electronics, with the detector output connected to its start input and the synchronism signal produced by the laser driver connected to the stop input. As the board presents a dead time of 100 ns after each photon detection, its saturated CR cannot overcome 10 Mcps (5 Mcps after considering the counting loss^10^). Consequently, the laser was operated at 4 MHz, making it possible to experimentally achieve a CR of 100% before pile-up correction without saturation. To obtain measurements with a SNR comparable with simulated DTOFs, the total acquisition time is increased to 10 s, thus acquiring curves equivalent to those that could have been obtained at 40 MHz. Therefore, all the CRs in the paper are reported considering this equivalence.

Measurements of homogeneous media are carried out using dedicated phantoms devised for the implementation of the MEDPHOT protocol^6^. 12 phantoms have been adopted for this work with ${\mu }_{a}$= 0.7, 0.13, 0.21 cm^−1^ and ${\mu }_{s}^{^{\prime}}$= 5, 8, 14, 20 cm^−1^ (conventionally true values at 690 nm), chosen to achieve all the targeted CRs (see Table 2). To prevent the overflow of counts in the histogram time bins, we acquire 100 repetitions of 100 ms. All repetitions are summed up to obtain DTOFs with the same SNR of simulations.

**Table 2:** CRs used for experimental study before (CR_sat,true_) and after (CR_true_) correction (first and fourth columns).

CR_sat,true_ [Mcps]	CR_sat_ [Mcps]	CR_sat_ [% Exc. rate]	CR_true_ [Mcps]	CR [Mcps]	CR [% Exc. rate]
0.125	1.245	3.11	0.13	1.265	3.16
2.529	25.285	63.21	4.00	40.000	100.00
3.324	33.243	83.11	7.11	71.131	177.83
3.831	38.307	95.77	12.65	126.491	316.23
3.986	39.856	99.64	22.50	224.937	562.34

Equivalent CRs obtained by summing up the 10 repetitions before (CR_sat_) and after (CR) correction (second and fifth columns). Percentages of the excitation rate before (CR_sat_ percentage) and after (CR percentage) correction (third and last columns).

Measurements of heterogeneous media are carried out using a switchable solid phantom devised for the implementation of the nEUROPt protocol^7^, composed by a bulk (${\mu }_{a}$= 0.1 cm^−1^ and ${\mu }_{s}^{^{\prime}}$= 10 cm^−1^) embedding a moving perturbation (a 0.1 cm^3^ black PVC cylinder, equivalent to a realistic inclusion of 1 cm^3^ volume with ${\Delta \mu }_{a}$ = 0.17 cm^−1^, at a depth of 1.5 cm from the top surface)^36^. We consider the same five CRs utilized for the MEDPHOT protocol, but we collect 1000 repetitions of 100 ms to end with 10 repetitions of curves acquired for 10 s as required for calculation of nEUROPt FOMs.

### Data analysis

The pile-up correction is applied to each simulated/acquired DTOF implementing the Coates’ algorithm^14^.

To evaluate the system capability to recover optical properties of homogenous medium, we adopt two of the tests stated in the MEDPHOT protocol: accuracy and linearity^6^.

Accuracy quantifies the instrument capability to recover the true value of ${\mu }_{a}$ and ${\mu }_{s}^{^{\prime}}$. The associated FOM is the relative error $\varepsilon$, as given in Eq. (2):

2 $$\varepsilon = \frac{{x - \tilde{x}}}{{\tilde{x}}}$$

where $x$ is the retrieved value (of either ${\mu }_{a}$ and ${\mu }_{s}^{^{\prime}}$) and $\tilde{x }$ is the conventionally true one.

Linearity, instead, assesses whether a system can linearly follow changes of ${\mu }_{a}$ and ${\mu }_{s}^{^{\prime}}$ without distortions and without crosstalk (i.e., coupling) between retrieved ${\mu }_{a}$ (${\mu }_{s}^{^{\prime}}$) and conventionally true ${\mu }_{s}^{^{\prime}}$ (${\mu }_{a}$). The associated FOMs are quantified in particular as the slope (SL) of the dependence between the conventionally true ($\tilde{x }$) and the retrieved ($x$) value of the considered optical property, as reported by Eq. (3):

3 $$x = SL \cdot \tilde{x} + q$$

where $SL$ can be either the linearity slope ($S{L}_{l}$, ideal value = 1, i.e., perfect linearity) or the crosstalk slope ($S{L}_{c}$, ideal value = 0, i.e., no crosstalk).

After background noise subtraction (when present, considering the background noise within 1–40 channel), we fitted the DTOFs (both before and after pile-up correction) with the same analytical model used for forward simulations. The fitting interval ranges from 20% of the peak on the rising edge down to 5% of the tail. The fitting algorithm converges and outputs the retrieved optical parameters when the χ^2^ (a goodness-of-fit merit function) is minimized via Levenberg-Marquardt method^43^ (used to compute MEDPHOT FOMs).

To evaluate the system capability in detecting a small absorption perturbation buried within homogenous medium, we use contrast (C) and contrast-to-noise ratio (CNR), i.e., the FOMs described in the nEUROPt protocol^7^.

The C quantifies the effect of the perturbation on the number of counts in the DTOF, it is computed as:

4 $$C = \frac{{\left\langle {N_{0} - N} \right\rangle }}{{\left\langle {N_{0} } \right\rangle }}$$

where $\left\langle {N_{0} - N} \right\rangle$ is the mean difference among the 10 repetitions between the number of counts inside a given time-gate along the DTOF temporal axis in the heterogenous ($N$) and homogenous (${N}_{0}$) case, while $\left\langle {N_{0} } \right\rangle$ is the average over repetitions of the number of counts in the homogeneous case.

The CNR is an index of the strength of the C against the noise of the measurement, and it is calculated as:

5 $$CNR = \frac{{\left\langle {N_{0} - N} \right\rangle }}{{\sigma \left( {N_{0} } \right)}}$$

where $\sigma \left({N}_{0}\right)$ represents the standard deviation of ${N}_{0}$ across the 10 repetitions.

Time gates for computing the nEUROPt FOMs are chosen as follows. Starting from the IRF peak, we employ 16 consecutive gates of 500 ps width along the DTOF temporal axis. Before computing C and CNR (both before and after correction), the background noise was subtracted (if present). Furthermore, we decide to add a visibility condition to the C. Indeed, C is computed only if the condition CNR ≥ 1 is satisfied within the gate.

Figure 5b,c summarize the whole simulation procedure from data generation to final analysis.

## Supplementary Information

Supplementary Information.

## Data Availability

The datasets generated and/or analysed during the current study are not publicly available due to the several and independent steps of data processing and their possible alternative choices (e.g., pile-up correction algorithm, background subtraction) but are available from the corresponding author on reasonable request.
