# Author Correction: Effects and correctability of pile‑up distortion using established figures of merit in time‑domain diffuse optics at extreme photon rates

**DOI:** 10.1038/s41598-022-14110-3

**Published:** 2022-06-08

**Authors:** Elisabetta Avanzi, Anurag Behera, Davide Contini, Lorenzo Spinelli, Alberto Dalla Mora, Laura Di Sieno

**Affiliations:** 1 Dipartimento di Fisica, Politecnico di Milano, Piazza Leonardo da Vinci 32, 20133 Milan, Italy; 2 Istituto di Fotonica e Nanotecnologie, Consiglio Nazionale delle Ricerche, Piazza Leonardo da Vinci 32, 20133 Milan, Italy

Correction to: *Scientific Reports* 10.1038/s41598-022-09385-5, published online 30 March 2022

The original version of this Article contained an error in Equation 1, where the sign of the ratio CR/$f_{laser}$ and the presence of the multiplier 100 was incorrect.

1 $$CR_{sat} = f_{laser} *\left( {1 - \exp \left( {\frac{CR}{{f_{laser} }}*100 } \right)} \right)$$

now reads:

1 $$CR_{sat} = f_{laser} *\left( {1 - \exp \left( { - \frac{CR}{{f_{laser} }} } \right)} \right)$$

The original Article has been corrected.

