# Supplementary material for: Effects and correctability of pile-up distortion using established figures of merit in time-domain diffuse optics at extreme photon rates
Source: Sci Rep. 2022 Mar 30;12:5417. doi: 10.1038/s41598-022-09385-5 (PMC8967884; doi:10.1038/s41598-022-09385-5)
Supplement: Supplementary file 1 — Supplementary Information. [file 41598_2022_9385_MOESM1_ESM.docx]

**Supplementary materials**

**Title:**

Effects and correctability of pile-up distortion using established figures of merit in time-domain diffuse optics at extreme photon rates

**Authors:**

Elisabetta Avanzi1, Anurag Behera1, Davide Contini1, Lorenzo Spinelli2,

Alberto Dalla Mora1, and Laura Di Sieno1

**Affiliations:**

^1^ Politecnico di Milano, Dipartimento di Fisica, Piazza Leonardo da Vinci 32, 20133 Milan, Italy

^2^ Consiglio Nazionale delle Ricerche, Istituto di Fotonica e Nanotecnologie, Piazza Leonardo da Vinci 32, 20133 Milan, Italy

**Corresponding author:**

[alberto.dallamora@polimi.it](mailto:alberto.dallamora@polimi.it)

| **CR [Mcps]** | **CR_sat_ [Mcps]** | **CR_sat_**  **[% Exc. rate]** | **μ_a_ accuracy**  **(%)** | **μ'_s_ accuracy**  **(%)** | **μ_a_ slope linearity** | **μ'_s_ slope linearity** | **μ_a_ over μ'_s_ slope crosstalk** | **μ'_s_ over μ_a_ slope crosstalk** |
| --- | --- | --- | --- | --- | --- | --- | --- | --- |
|  |  |  | **BEFORE CORRECTION** | | | | | |
| 0.400 | 0.398 | 1.00 | 0.18 ± 0.44 | 0.06 ± 0.39 | 1.000 ± 0.004 | 1.001 ± 0.007 | 0.0000 ± 0.0002 | -0.0416 ± 0.1997 |
| 0.711 | 0.705 | 1.76 | 0.09 ± 0.49 | -0.16 ± 0.42 | 1.001 ± 0.004 | 0.997 ± 0.006 | 0.0000 ± 0.0000 | -0.0405 ± 0.1738 |
| 1.265 | 1.245 | 3.11 | 0.26 ± 0.32 | -0.32 ± 0.31 | 1.004 ± 0.002 | 0.994 ± 0.005 | -0.0001 ± 0.0001 | 0.2204 ± 0.1270 |
| 2.249 | 2.187 | 5.47 | 0.34 ± 0.39 | -0.78 ± 0.21 | 1.000 ± 0.006 | 0.990 ± 0.004 | -0.0001 ± 0.0001 | -0.0042 ± 0.1512 |
| 4.000 | 3.807 | 9.52 | 0.34 ± 0.37 | -1.74 ± 0.20 | 0.999 ± 0.003 | 0.984 ± 0.001 | -0.0001 ± 0.0001 | 0.1129 ± 0.0198 |
| 7.113 | 6.517 | 16.29 | 0.56 ± 0.67 | -3.30 ± 0.27 | 0.998 ± 0.002 | 0.969 ± 0.004 | -0.0002 ± 0.0000 | 0.1779 ± 0.2025 |
| 12.649 | 10.844 | 27.11 | 1.31 ± 1.22 | -5.72 ± 0.32 | 0.999 ± 0.008 | 0.946 ± 0.003 | -0.0004 ± 0.0002 | 0.2060 ± 0.1536 |
| 22.494 | 17.205 | 43.01 | 3.46 ± 2.38 | -9.33 ± 0.50 | 1.005 ± 0.014 | 0.913 ± 0.001 | -0.0007 ± 0.0003 | 0.2322 ± 0.0652 |
| 40.000 | 25.285 | 63.21 | 10.14 ± 4.87 | -13.54 ± 0.86 | 1.042 ± 0.024 | 0.876 ± 0.006 | -0.0013 ± 0.0005 | 0.4928 ± 0.1977 |
| 71.131 | 33.243 | 83.11 | 31.82 ± 10.76 | -14.69 ± 1.46 | 1.185 ± 0.047 | 0.870 ± 0.008 | -0.0029 ± 0.0009 | 0.9803 ± 0.4548 |
| 126.491 | 38.307 | 95.77 | 102.34 ± 26.99 | -0.75 ± 2.89 | 1.681 ± 0.094 | 1.023 ± 0.020 | -0.0068 ± 0.0018 | 2.4701 ± 0.9987 |
| 224.937 | 39.856 | 99.64 | 275.63 ± 63.41 | 38.92 ± 7.67 | 2.938 ± 0.181 | 1.474 ± 0.069 | -0.0151 ± 0.0035 | 6.8223 ± 3.3731 |
| 400.000 | 39.998 | 100.00 | 563.44 ± 132.87 | 93.04 ± 13.69 | 4.888 ± 0.324 | 2.094 ± 0.128 | -0.0305 ± 0.0063 | 11.9779 ± 6.2528 |
|  |  |  | **AFTER CORRECTION** | | | | | |
| 0.400 | 0.398 | 1.00 | 0.18±0.42 | 0.28±0.37 | 1.000±0.002 | 1.004±0.005 | 0.00004±0.00010 | -0.00965±0.06107 |
| 0.711 | 0.705 | 1.76 | 0.08±0.46 | 0.24±0.40 | 1.003±0.004 | 1.001±0.006 | 0.00000±0.00003 | -0.00150±0.21339 |
| 1.265 | 1.245 | 3.11 | 0.20±0.27 | 0.34±0.31 | 1.005±0.001 | 1.000±0.004 | -0.00003±0.00007 | 0.23825±0.14315 |
| 2.249 | 2.187 | 5.47 | 0.24±0.28 | 0.41±0.27 | 1.002±0.005 | 1.001±0.004 | -0.00008±0.00012 | -0.00627±0.14436 |
| 4.000 | 3.807 | 9.52 | 0.13±0.17 | 0.32±0.19 | 1.002±0.001 | 1.003±0.001 | -0.00001±0.00001 | 0.08025±0.04001 |
| 7.113 | 6.517 | 16.29 | 0.09±0.18 | 0.28±0.21 | 1.001±0.003 | 1.001±0.003 | 0.00002±0.00005 | 0.13256±0.17947 |
| 12.649 | 10.844 | 27.11 | 0.11±0.14 | 0.32±0.19 | 1.002±0.002 | 1.001±0.001 | -0.00002±0.00003 | 0.09649±0.08657 |
| 22.494 | 17.205 | 43.01 | 0.12±0.09 | 0.32±0.14 | 1.001±0.001 | 1.001±0.001 | -0.00002±0.00003 | 0.04187±0.01744 |
| 40.000 | 25.285 | 63.21 | 0.16±0.10 | 0.35±0.17 | 1.002±0.001 | 1.002±0.002 | -0.00002±0.00002 | 0.11228±0.08031 |
| 71.131 | 33.243 | 83.11 | 0.17±0.07 | 0.35±0.11 | 1.002±0.001 | 1.002±0.000 | 0.00000±0.00000 | 0.06671±0.04284 |
| 126.491 | 38.307 | 95.77 | 0.14±0.17 | 0.34±0.19 | 1.004±0.002 | 1.002±0.002 | -0.00002±0.00006 | 0.09276±0.01717 |
| 224.937 | 39.856 | 99.64 | 0.21±0.29 | 0.35±0.21 | 0.999±0.003 | 1.002±0.004 | 0.00000±0.00013 | 0.01520±0.08515 |
| 400.000 | 39.998 | 100.00 | 2.84±2.68 | 2.13±1.60 | 0.986±0.018 | 1.040±0.010 | 0.00051±0.00035 | -1.16109±0.49754 |

**Table S1** MEDPHOT results for delta-Dirac IRF simulations.

| **CR [Mcps]** | **CR_sat_ [Mcps]** | **CR_sat_**  **[% Exc. rate]** | **μ_a_ accuracy**  **(%)** | **μ'_s_ accuracy**  **(%)** | **μ_a_ slope linearity** | **μ'_s_ slope linearity** | **μ_a_ over μ'_s_ slope crosstalk** | **μ'_s_ over μ_a_ slope crosstalk** |
| --- | --- | --- | --- | --- | --- | --- | --- | --- |
|  |  |  | **BEFORE CORRECTION** | | | | | |
| 0.400 | 0.398 | 1.00 | -25.54±6.40 | -24.12±3.87 | 0.835 ± 0.015 | 0.721 ± 0.064 | -0.0002 ± 0.0004 | 2.975 ± 2.846 |
| 0.711 | 0.705 | 1.76 | -11.73±2.78 | -11.13±1.39 | 0.926 ± 0.015 | 0.889 ± 0.030 | 0.0002 ± 0.0003 | 1.364 ± 1.349 |
| 1.265 | 1.245 | 3.11 | -5.22±1.89 | -5.52±1.68 | 0.961 ± 0.018 | 0.930 ± 0.024 | -0.0002 ± 0.0003 | 0.502 ± 1.122 |
| 2.249 | 2.187 | 5.47 | -2.35±1.05 | -3.53±1.00 | 0.986 ± 0.005 | 0.962 ± 0.014 | -0.0001 ± 0.0002 | 0.283 ± 0.585 |
| 4.000 | 3.807 | 9.52 | -0.93±0.43 | -3.58±0.46 | 0.995 ± 0.005 | 0.969 ± 0.003 | -0.0001 ± 0.0001 | 0.157 ± 0.179 |
| 7.113 | 6.517 | 16.29 | 0.44±0.59 | -4.61±0.80 | 1.012 ± 0.011 | 0.965 ± 0.001 | -0.0002 ± 0.0002 | 0.299 ± 0.126 |
| 12.649 | 10.844 | 27.11 | 2.18±0.74 | -6.98±1.34 | 1.028 ± 0.012 | 0.953 ± 0.002 | -0.0002 ± 0.0002 | 0.312 ± 0.088 |
| 22.494 | 17.205 | 43.01 | 5.37±1.84 | -11.13±2.10 | 1.067 ± 0.036 | 0.921 ± 0.004 | -0.0007 ± 0.0007 | 0.530 ± 0.137 |
| 40.000 | 25.285 | 63.21 | 13.91±5.03 | -16.16±3.71 | 1.189 ± 0.098 | 0.883 ± 0.004 | -0.0019 ± 0.0019 | 1.732 ± 0.138 |
| 71.131 | 33.243 | 83.11 | 46.74±25.07 | -13.86±13.45 | 1.799 ± 0.500 | 0.856 ± 0.010 | -0.0085 ± 0.0098 | 8.462 ± 0.706 |
| 126.491 | 38.307 | 95.77 | 391.47±572.23 | 127.26±264.46 | 11.942 ± 2.938 | 0.248 ± 1.010 | -0.1793 ± 0.2472 | 98.949 ± 51.513 |
| 224.937 | 39.856 | 99.64 | 1955.72±1228.39 | 594.74±438.96 | 30.384 ± 6.159 | 5.854 ± 7.172 | -0.2157 ± 0.2522 | 368.068 ± 331.363 |
| 400.000 | 39.998 | 100.00 | 2427.45±1956.72 | 549.75±573.04 | 8.612 ± 14.138 | 12.341 ± 8.295 | 0.4027 ± 0.2841 | -142.366 ± 215.480 |
|  |  |  | **AFTER CORRECTION** | | | | | |
| 0.400 | 0.398 | 1.00 | -25.70 ± 6.37 | -24.11 ± 3.84 | 0.832 ± 0.014 | 0.718 ± 0.060 | -0.0002 ± 0.0004 | 2.808 ± 2.547 |
| 0.711 | 0.705 | 1.76 | -11.94 ± 2.82 | -10.92 ± 1.39 | 0.924 ± 0.015 | 0.890 ± 0.030 | 0.0002 ± 0.0003 | 1.345 ± 1.329 |
| 1.265 | 1.245 | 3.11 | -6.05 ± 1.51 | -5.50 ± 0.89 | 0.963 ± 0.007 | 0.939 ± 0.014 | 0.0000 ± 0.0002 | 0.751 ± 0.707 |
| 2.249 | 2.187 | 5.47 | -3.12 ± 1.04 | -2.78 ± 0.73 | 0.984 ± 0.009 | 0.973 ± 0.008 | 0.0000 ± 0.0001 | 0.493 ± 0.465 |
| 4.000 | 3.807 | 9.52 | -1.60 ± 0.53 | -1.39 ± 0.54 | 0.986 ± 0.005 | 0.980 ± 0.005 | -0.0001 ± 0.0001 | 0.055 ± 0.293 |
| 7.113 | 6.517 | 16.29 | -0.88 ± 0.35 | -0.77 ± 0.37 | 0.996 ± 0.002 | 0.989 ± 0.004 | -0.0001 ± 0.0000 | 0.174 ± 0.203 |
| 12.649 | 10.844 | 27.11 | -0.49 ± 0.23 | -0.45 ± 0.24 | 0.997 ± 0.004 | 0.998 ± 0.004 | 0.0001 ± 0.0001 | 0.089 ± 0.202 |
| 22.494 | 17.205 | 43.01 | -0.31 ± 0.16 | -0.29 ± 0.21 | 0.997 ± 0.001 | 0.996 ± 0.001 | 0.0000 ± 0.0000 | -0.023 ± 0.036 |
| 40.000 | 25.285 | 63.21 | -0.13 ± 0.13 | -0.12 ± 0.14 | 0.999 ± 0.002 | 0.998 ± 0.002 | 0.0000 ± 0.0000 | -0.001 ± 0.047 |
| 71.131 | 33.243 | 83.11 | -0.03 ± 0.09 | -0.03 ± 0.10 | 0.999 ± 0.001 | 1.000 ± 0.001 | 0.0000 ± 0.0000 | -0.042 ± 0.034 |
| 126.491 | 38.307 | 95.77 | -0.05 ± 0.15 | -0.05 ± 0.13 | 0.999 ± 0.003 | 1.000 ± 0.001 | 0.0000 ± 0.0000 | -0.030 ± 0.049 |
| 224.937 | 39.856 | 99.64 | 0.16 ± 0.25 | 0.12 ± 0.19 | 1.000 ± 0.002 | 1.000 ± 0.001 | 0.0000 ± 0.0001 | -0.052 ± 0.132 |
| 400.000 | 39.998 | 100.00 | 1049.57 ± 1128.98 | 864.15 ± 919.53 | 8.508 ± 16.905 | 18.765 ± 10.046 | 0.2356 ± 0.2651 | -6.761 ± 679.571 |

**Table S2** MEDPHOT results for SiPM-like IRF simulations.

| **CR* [Mcps]** | **CR_sat_* [Mcps]** | **CR_sat_**  **[% Exc. rate]** | **μ_a_ accuracy**  **(%)** | **μ'_s_ accuracy**  **(%)** | **μ_a_ slope linearity** | **μ'_s_ slope linearity** | **μ_a_ over μ'_s_ slope crosstalk** | **μ'_s_ over μ_a_ slope crosstalk** |
| --- | --- | --- | --- | --- | --- | --- | --- | --- |
|  |  |  | **BEFORE CORRECTION** | | | | | |
| 1.265 | 1.245 | 3.11 | -6.17 ± 15.61 | 5.94 ± 13.81 | 0.925 ± 0.221 | 0.955 ± 0.276 | 0.0002 ± 0.0003 | -0.239 ± 4.924 |
| 40.000 | 25.285 | 63.21 | 11.97 ± 7.86 | -7.54 ± 8.47 | 1.078 ± 0.039 | 0.909 ± 0.046 | -0.0004 ± 0.0005 | 3.098 ± 5.030 |
| 71.131 | 33.243 | 83.11 | 42.86 ± 21.37 | -7.83 ± 9.96 | 1.218 ± 0.066 | 0.889 ± 0.057 | -0.0013 ± 0.0013 | 8.164 ± 7.066 |
| 126.491 | 38.307 | 95.77 | 163.64 ± 79.19 | 12.35 ± 19.04 | 1.900 ± 0.132 | 1.195 ± 0.303 | -0.0072 ± 0.0080 | 31.889 ± 26.890 |
| 224.937 | 39.856 | 99.64 | 539.27 ± 307.45 | 118.41 ± 60.86 | 3.819 ± 0.335 | 2.100 ± 0.868 | -0.0207 ± 0.0211 | 88.574 ± 43.158 |
|  |  |  | **AFTER CORRECTION** | | | | | |
| 1.265 | 1.245 | 3.11 | -6.20 ± 15.57 | 6.31 ± 13.86 | 0.925 ± 0.221 | 0.957 ± 0.277 | 0.0003 ± 0.0003 | -0.305 ± 4.981 |
| 40.000 | 25.285 | 63.21 | -0.89 ± 11.40 | 8.10 ± 8.68 | 1.037 ± 0.028 | 1.074 ± 0.054 | 0.0003 ± 0.0002 | -0.774 ± 4.979 |
| 71.131 | 33.243 | 83.11 | 2.22 ± 10.93 | 11.00 ± 9.21 | 1.054 ± 0.020 | 1.087 ± 0.055 | 0.0002 ± 0.0002 | 0.024 ± 5.203 |
| 126.491 | 38.307 | 95.77 | 3.59 ± 12.74 | 12.10 ± 11.57 | 1.093 ± 0.036 | 1.241 ± 0.188 | -0.0002 ± 0.0011 | 0.417 ± 2.014 |
| 224.937 | 39.856 | 99.64 | 6.73 ± 10.55 | 17.39 ± 11.76 | 1.072 ± 0.050 | 1.300 ± 0.242 | 0.0001 ± 0.0004 | -1.869 ± 4.677 |

**Table S3** MEDPHOT results for measurements.

* Note that the CR and CR_sat_ here reported are obtained by summing up 10 repetitions of 1 s, thus acquiring curves equivalent to those that could have been obtained at 40 MHz

| **Contrast** | | | | | | | | | | | | | | | | | | |
| --- | --- | --- | --- | --- | --- | --- | --- | --- | --- | --- | --- | --- | --- | --- | --- | --- | --- | --- |
| **CR [Mcps]** | **CR_sat_ [Mcps]** | **CR_sat_**  **[% Exc. rate]** | **0-0.5 ns** | **0.5-1.0 ns** | **1.0-1.5 ns** | **1.5-2.0 ns** | **2.0-2.5 ns** | **2.5-3.0 ns** | **3.0-3.5 ns** | **3.5-4.0 ns** | **4.0-4.5 ns** | **4.5-5.0 ns** | **5.0-5.5 ns** | **5.5-6.0 ns** | **6.0-6.5 ns** | **6.5-7.0 ns** | **7.0-7.5 ns** | **7.5-8.0 ns** |
|  |  |  | **BEFORE CORRECTION** | | | | | | | | | | | | | | | |
| 0.400 | 0.398 | 1.00 | NaN | 0.029 | 0.110 | 0.200 | 0.269 | 0.325 | 0.336 | 0.371 | 0.393 | 0.338 | 0.413 | NaN | 0.217 | NaN | NaN | NaN |
| 0.711 | 0.705 | 1.76 | NaN | 0.031 | 0.108 | 0.199 | 0.268 | 0.315 | 0.343 | 0.363 | 0.372 | 0.408 | 0.410 | 0.409 | NaN | NaN | NaN | NaN |
| 1.265 | 1.245 | 3.11 | NaN | 0.030 | 0.108 | 0.197 | 0.268 | 0.319 | 0.337 | 0.357 | 0.353 | 0.313 | 0.397 | NaN | NaN | NaN | NaN | NaN |
| 2.249 | 2.187 | 5.47 | NaN | 0.030 | 0.106 | 0.197 | 0.266 | 0.313 | 0.338 | 0.364 | 0.368 | 0.361 | 0.339 | 0.279 | 0.416 | NaN | NaN | NaN |
| 4.000 | 3.807 | 9.52 | NaN | 0.030 | 0.105 | 0.195 | 0.263 | 0.309 | 0.338 | 0.365 | 0.364 | 0.391 | 0.382 | 0.408 | 0.310 | NaN | NaN | NaN |
| 7.113 | 6.517 | 16.29 | NaN | 0.029 | 0.103 | 0.191 | 0.259 | 0.303 | 0.335 | 0.353 | 0.360 | 0.358 | 0.356 | 0.402 | 0.336 | 0.378 | NaN | NaN |
| 12.649 | 10.844 | 27.11 | NaN | 0.028 | 0.099 | 0.183 | 0.251 | 0.296 | 0.327 | 0.341 | 0.348 | 0.355 | 0.327 | 0.386 | 0.350 | 0.334 | NaN | NaN |
| 22.494 | 17.205 | 43.01 | NaN | 0.027 | 0.092 | 0.170 | 0.235 | 0.280 | 0.309 | 0.328 | 0.333 | 0.353 | 0.332 | 0.297 | 0.282 | 0.314 | NaN | NaN |
| 40.000 | 25.285 | 63.21 | 0.002 | 0.024 | 0.080 | 0.147 | 0.206 | 0.252 | 0.280 | 0.301 | 0.311 | 0.313 | 0.324 | 0.321 | 0.219 | 0.196 | NaN | NaN |
| 71.131 | 33.243 | 83.11 | 0.002 | 0.020 | 0.059 | 0.104 | 0.153 | 0.196 | 0.226 | 0.245 | 0.249 | 0.260 | 0.245 | 0.273 | 0.247 | 0.184 | NaN | NaN |
| 126.491 | 38.307 | 95.77 | 0.002 | 0.015 | 0.024 | 0.024 | 0.049 | 0.088 | 0.118 | 0.143 | 0.152 | 0.172 | 0.146 | 0.126 | NaN | NaN | NaN | NaN |
| 224.937 | 39.856 | 99.64 | 0.001 | 0.007 | NaN | NaN | NaN | NaN | NaN | NaN | NaN | NaN | NaN | NaN | NaN | NaN | NaN | NaN |
| 400.000 | 39.998 | 100.00 | 0.001 | 0.002 | NaN | NaN | NaN | NaN | NaN | NaN | NaN | NaN | NaN | NaN | NaN | NaN | NaN | NaN |
|  |  |  | **AFTER CORRECTION** | | | | | | | | | | | | | | | |
| 0.400 | 0.398 | 1.00 | NaN | 0.029 | 0.110 | 0.200 | 0.270 | 0.326 | 0.337 | 0.371 | 0.394 | 0.338 | 0.414 | NaN | 0.217 | NaN | NaN | NaN |
| 0.711 | 0.705 | 1.76 | NaN | 0.031 | 0.108 | 0.200 | 0.269 | 0.316 | 0.344 | 0.364 | 0.373 | 0.409 | 0.411 | 0.410 | NaN | NaN | NaN | NaN |
| 1.265 | 1.245 | 3.11 | NaN | 0.031 | 0.109 | 0.199 | 0.270 | 0.321 | 0.339 | 0.359 | 0.355 | 0.315 | 0.399 | NaN | NaN | NaN | NaN | NaN |
| 2.249 | 2.187 | 5.47 | NaN | 0.031 | 0.108 | 0.200 | 0.269 | 0.316 | 0.341 | 0.368 | 0.371 | 0.364 | 0.342 | 0.283 | 0.419 | NaN | NaN | NaN |
| 4.000 | 3.807 | 9.52 | NaN | 0.030 | 0.108 | 0.200 | 0.269 | 0.315 | 0.344 | 0.371 | 0.370 | 0.397 | 0.388 | 0.414 | 0.317 | NaN | NaN | NaN |
| 7.113 | 6.517 | 16.29 | NaN | 0.030 | 0.108 | 0.200 | 0.270 | 0.315 | 0.346 | 0.363 | 0.371 | 0.369 | 0.367 | 0.413 | 0.347 | 0.389 | NaN | NaN |
| 12.649 | 10.844 | 27.11 | NaN | 0.030 | 0.107 | 0.199 | 0.270 | 0.316 | 0.347 | 0.361 | 0.367 | 0.374 | 0.347 | 0.405 | 0.370 | 0.354 | NaN | NaN |
| 22.494 | 17.205 | 43.01 | NaN | 0.031 | 0.108 | 0.199 | 0.270 | 0.316 | 0.345 | 0.363 | 0.367 | 0.387 | 0.367 | 0.334 | 0.319 | 0.350 | NaN | NaN |
| 40.000 | 25.285 | 63.21 | 0.002 | 0.030 | 0.108 | 0.199 | 0.269 | 0.318 | 0.344 | 0.365 | 0.374 | 0.376 | 0.386 | 0.383 | 0.290 | 0.269 | NaN | NaN |
| 71.131 | 33.243 | 83.11 | 0.002 | 0.030 | 0.108 | 0.199 | 0.270 | 0.316 | 0.345 | 0.362 | 0.366 | 0.375 | 0.363 | 0.387 | 0.365 | 0.312 | 0.232 | NaN |
| 126.491 | 38.307 | 95.77 | 0.002 | 0.031 | 0.108 | 0.200 | 0.269 | 0.316 | 0.345 | 0.365 | 0.373 | 0.388 | 0.368 | 0.354 | 0.320 | NaN | NaN | NaN |
| 224.937 | 39.856 | 99.64 | 0.001 | 0.031 | 0.108 | 0.200 | 0.270 | 0.317 | 0.348 | 0.367 | 0.346 | 0.408 | 0.390 | 0.187 | NaN | NaN | NaN | NaN |
| 400.000 | 39.998 | 100.00 | 0.001 | 0.031 | 0.109 | 0.206 | 0.255 | 0.331 | 0.360 | 0.316 | NaN | NaN | Inf | NaN | NaN | NaN | NaN | NaN |

**Table S4** nEUROPt results (only contrast) for delta-Dirac IRF simulations.

| **Contrast-to-Noise Ratio** | | | | | | | | | | | | | | | | | | |
| --- | --- | --- | --- | --- | --- | --- | --- | --- | --- | --- | --- | --- | --- | --- | --- | --- | --- | --- |
| **CR [Mcps]** | **CR_sat_ [Mcps]** | **CR_sat_**  **[% Exc. rate]** | **0-0.5 ns** | **0.5-1.0 ns** | **1.0-1.5 ns** | **1.5-2.0 ns** | **2.0-2.5 ns** | **2.5-3.0 ns** | **3.0-3.5 ns** | **3.5-4.0 ns** | **4.0-4.5 ns** | **4.5-5.0 ns** | **5.0-5.5 ns** | **5.5-6.0 ns** | **6.0-6.5 ns** | **6.5-7.0 ns** | **7.0-7.5 ns** | **7.5-8.0 ns** |
|  |  |  | **BEFORE CORRECTION** | | | | | | | | | | | | | | | |
| 0.400 | 0.398 | 1.00 | 0.48 | 15.73 | 44.52 | 66.81 | 40.76 | 28.84 | 14.95 | 6.19 | 7.18 | 2.77 | 1.95 | 0.51 | 1.07 | 0.22 | 0.82 | -Inf |
| 0.711 | 0.705 | 1.76 | 0.32 | 15.74 | 51.23 | 70.61 | 54.03 | 32.01 | 28.22 | 16.21 | 5.76 | 6.56 | 3.04 | 1.80 | 0.51 | 0.15 | 0.44 | -Inf |
| 1.265 | 1.245 | 3.11 | 0.34 | 26.34 | 91.77 | 64.14 | 71.38 | 56.57 | 24.43 | 21.60 | 7.89 | 4.18 | 6.47 | 0.95 | 0.74 | 0.10 | 0.38 | 0.33 |
| 2.249 | 2.187 | 5.47 | 0.73 | 31.44 | 131.07 | 85.75 | 101.17 | 75.95 | 42.31 | 15.90 | 13.88 | 5.46 | 3.05 | 2.54 | 1.68 | 0.00 | 0.34 | -0.22 |
| 4.000 | 3.807 | 9.52 | 0.00 | 51.49 | 143.77 | 116.29 | 95.99 | 110.49 | 61.45 | 21.79 | 19.73 | 6.81 | 4.61 | 3.17 | 1.63 | 0.79 | 0.20 | 0.00 |
| 7.113 | 6.517 | 16.29 | 0.94 | 42.45 | 247.85 | 174.19 | 140.57 | 89.12 | 67.77 | 33.14 | 22.09 | 10.25 | 6.61 | 4.52 | 1.83 | 1.42 | 0.32 | 0.38 |
| 12.649 | 10.844 | 27.11 | 0.27 | 89.14 | 204.43 | 230.07 | 229.95 | 95.86 | 97.54 | 34.15 | 20.80 | 9.87 | 8.71 | 4.17 | 5.46 | 1.34 | 0.60 | 0.17 |
| 22.494 | 17.205 | 43.01 | 0.42 | 77.54 | 249.16 | 270.03 | 148.80 | 110.84 | 46.48 | 49.84 | 20.25 | 22.79 | 11.51 | 3.71 | 2.36 | 1.36 | 0.62 | 0.14 |
| 40.000 | 25.285 | 63.21 | 2.59 | 121.17 | 274.65 | 261.62 | 187.83 | 132.05 | 84.17 | 47.71 | 21.27 | 16.55 | 6.38 | 5.79 | 1.54 | 1.16 | -0.08 | 0.56 |
| 71.131 | 33.243 | 83.11 | 4.00 | 285.53 | 250.71 | 206.96 | 145.52 | 75.83 | 84.53 | 64.45 | 20.75 | 11.18 | 5.48 | 3.40 | 2.10 | 1.10 | 0.88 | 0.38 |
| 126.491 | 38.307 | 95.77 | 3.44 | 110.63 | 52.81 | 37.63 | 28.29 | 21.93 | 15.28 | 10.71 | 8.52 | 8.22 | 3.38 | 1.39 | 0.66 | 0.32 | 0.14 | -0.30 |
| 224.937 | 39.856 | 99.64 | 4.00 | 108.41 | -69.65 | -69.82 | -25.85 | -15.90 | -8.16 | -2.48 | -2.08 | -0.14 | -0.31 | -1.33 | 0.05 | 0.13 | -0.22 | 0.40 |
| 400.000 | 39.998 | 100.00 | 5.53 | 17.63 | -133.47 | -59.25 | -28.82 | -10.92 | -5.83 | -2.57 | -2.52 | -1.34 | 0.37 | -0.67 | 0.00 | NaN | NaN | NaN |
|  |  |  | **AFTER CORRECTION** | | | | | | | | | | | | | | | |
| 0.400 | 0.398 | 1.00 | 0.48 | 15.72 | 44.58 | 66.94 | 40.82 | 28.88 | 14.98 | 6.20 | 7.19 | 2.77 | 1.95 | 0.51 | 1.07 | 0.22 | 0.82 | -Inf |
| 0.711 | 0.705 | 1.76 | 0.32 | 15.74 | 51.13 | 70.56 | 54.14 | 32.09 | 28.31 | 16.25 | 5.77 | 6.58 | 3.04 | 1.81 | 0.51 | 0.15 | 0.44 | -Inf |
| 1.265 | 1.245 | 3.11 | 0.34 | 26.32 | 91.65 | 64.37 | 71.90 | 56.93 | 24.57 | 21.74 | 7.93 | 4.21 | 6.50 | 0.96 | 0.75 | 0.10 | 0.38 | 0.33 |
| 2.249 | 2.187 | 5.47 | 0.73 | 31.43 | 132.54 | 86.55 | 102.32 | 76.39 | 42.72 | 16.05 | 14.01 | 5.51 | 3.08 | 2.57 | 1.69 | 0.01 | 0.35 | -0.21 |
| 4.000 | 3.807 | 9.52 | 0.00 | 51.32 | 144.51 | 116.83 | 97.90 | 112.43 | 62.37 | 22.10 | 20.06 | 6.91 | 4.68 | 3.22 | 1.66 | 0.80 | 0.21 | 0.00 |
| 7.113 | 6.517 | 16.29 | 0.94 | 42.25 | 233.66 | 184.58 | 146.23 | 92.48 | 70.31 | 34.27 | 22.72 | 10.56 | 6.81 | 4.63 | 1.88 | 1.45 | 0.34 | 0.39 |
| 12.649 | 10.844 | 27.11 | 0.27 | 85.72 | 198.57 | 246.88 | 248.32 | 100.76 | 103.58 | 35.84 | 21.93 | 10.39 | 9.25 | 4.36 | 5.76 | 1.40 | 0.65 | 0.20 |
| 22.494 | 17.205 | 43.01 | 0.42 | 77.78 | 319.40 | 329.03 | 168.25 | 126.76 | 51.77 | 55.00 | 22.31 | 25.00 | 12.73 | 4.16 | 2.66 | 1.48 | 0.71 | 0.17 |
| 40.000 | 25.285 | 63.21 | 2.59 | 118.09 | 336.17 | 330.17 | 232.00 | 159.24 | 102.91 | 58.15 | 25.67 | 19.94 | 7.58 | 6.88 | 1.97 | 1.54 | 0.14 | 0.67 |
| 71.131 | 33.243 | 83.11 | 4.00 | 295.90 | 444.32 | 396.79 | 262.14 | 122.65 | 129.39 | 94.39 | 30.75 | 16.14 | 8.09 | 4.78 | 3.06 | 1.56 | 1.12 | 0.49 |
| 126.491 | 38.307 | 95.77 | 3.44 | 120.11 | 424.50 | 255.93 | 170.73 | 72.94 | 46.26 | 27.84 | 20.99 | 18.52 | 8.43 | 3.73 | 1.70 | 0.91 | 0.37 | 0.01 |
| 224.937 | 39.856 | 99.64 | 4.00 | 130.72 | 154.05 | 74.53 | 59.07 | 44.09 | 28.03 | 10.87 | 6.16 | 5.74 | 3.48 | 1.13 | 0.99 | 0.61 | 0.14 | 0.65 |
| 400.000 | 39.998 | 100.00 | 5.52 | 208.71 | 38.82 | 17.67 | 14.39 | 5.14 | 3.90 | 1.49 | 0.80 | 0.16 | 1.16 | -0.06 | 0.20 | NaN | NaN | NaN |

**Table S5** nEUROPt results (only Contrast-to-Noise Ratio) for delta-Dirac IRF simulations.

| **Contrast** | | | | | | | | | | | | | | | | | | |
| --- | --- | --- | --- | --- | --- | --- | --- | --- | --- | --- | --- | --- | --- | --- | --- | --- | --- | --- |
| **CR [Mcps]** | **CR_sat_ [Mcps]** | **CR_sat_**  **[% Exc. rate]** | **0-0.5 ns** | **0.5-1.0 ns** | **1.0-1.5 ns** | **1.5-2.0 ns** | **2.0-2.5 ns** | **2.5-3.0 ns** | **3.0-3.5 ns** | **3.5-4.0 ns** | **4.0-4.5 ns** | **4.5-5.0 ns** | **5.0-5.5 ns** | **5.5-6.0 ns** | **6.0-6.5 ns** | **6.5-7.0 ns** | **7.0-7.5 ns** | **7.5-8.0 ns** |
|  |  |  | **BEFORE CORRECTION** | | | | | | | | | | | | | | | |
| 0.400 | 0.398 | 1.00 | NaN | 0.024 | 0.083 | 0.159 | 0.218 | 0.238 | 0.206 | 0.141 | 0.123 | NaN | 0.093 | NaN | NaN | NaN | NaN | NaN |
| 0.711 | 0.705 | 1.76 | NaN | 0.025 | 0.082 | 0.157 | 0.215 | 0.235 | 0.212 | 0.168 | 0.132 | 0.129 | NaN | NaN | NaN | NaN | NaN | NaN |
| 1.265 | 1.245 | 3.11 | NaN | 0.025 | 0.082 | 0.158 | 0.217 | 0.234 | 0.215 | 0.179 | 0.149 | 0.124 | 0.138 | 0.128 | 0.143 | 0.152 | 0.154 | 0.200 |
| 2.249 | 2.187 | 5.47 | 0.005 | 0.024 | 0.081 | 0.157 | 0.214 | 0.232 | 0.211 | 0.159 | 0.126 | 0.112 | 0.119 | 0.110 | 0.121 | 0.101 | 0.106 | 0.122 |
| 4.000 | 3.807 | 9.52 | NaN | 0.023 | 0.081 | 0.155 | 0.213 | 0.228 | 0.203 | 0.155 | 0.119 | 0.099 | 0.094 | 0.092 | 0.086 | 0.075 | 0.096 | 0.084 |
| 7.113 | 6.517 | 16.29 | 0.003 | 0.024 | 0.078 | 0.152 | 0.208 | 0.224 | 0.198 | 0.152 | 0.116 | 0.103 | 0.090 | 0.084 | 0.085 | 0.080 | 0.075 | 0.085 |
| 12.649 | 10.844 | 27.11 | 0.002 | 0.023 | 0.076 | 0.147 | 0.201 | 0.217 | 0.190 | 0.148 | 0.112 | 0.091 | 0.084 | 0.088 | 0.086 | 0.084 | 0.078 | 0.083 |
| 22.494 | 17.205 | 43.01 | 0.003 | 0.022 | 0.071 | 0.136 | 0.188 | 0.202 | 0.176 | 0.126 | 0.091 | 0.068 | 0.065 | 0.056 | 0.061 | 0.062 | 0.053 | 0.055 |
| 40.000 | 25.285 | 63.21 | 0.002 | 0.021 | 0.062 | 0.117 | 0.164 | 0.175 | 0.145 | 0.097 | 0.057 | 0.037 | 0.028 | 0.024 | 0.026 | 0.019 | 0.022 | 0.011 |
| 71.131 | 33.243 | 83.11 | 0.002 | 0.018 | 0.047 | 0.084 | 0.119 | 0.124 | 0.091 | 0.033 | NaN | NaN | NaN | NaN | NaN | NaN | NaN | NaN |
| 126.491 | 38.307 | 95.77 | 0.002 | 0.013 | 0.022 | 0.022 | 0.034 | 0.028 | NaN | NaN | NaN | NaN | NaN | NaN | NaN | NaN | NaN | NaN |
| 224.937 | 39.856 | 99.64 | 0.002 | 0.007 | NaN | NaN | NaN | NaN | NaN | NaN | NaN | NaN | NaN | NaN | NaN | NaN | NaN | NaN |
| 400.000 | 39.998 | 100.00 | 0.002 | 0.002 | NaN | NaN | NaN | NaN | NaN | NaN | NaN | NaN | NaN | NaN | NaN | NaN | NaN | NaN |
|  |  |  | **AFTER CORRECTION** | | | | | | | | | | | | | | | |
| 0.400 | 0.398 | 1.00 | NaN | 0.024 | 0.084 | 0.159 | 0.219 | 0.238 | 0.206 | 0.141 | 0.123 | NaN | 0.094 | NaN | NaN | NaN | NaN | NaN |
| 0.711 | 0.705 | 1.76 | NaN | 0.025 | 0.082 | 0.158 | 0.215 | 0.235 | 0.212 | 0.169 | 0.132 | 0.130 | NaN | NaN | 0.110 | NaN | NaN | NaN |
| 1.265 | 1.245 | 3.11 | NaN | 0.025 | 0.083 | 0.159 | 0.218 | 0.235 | 0.216 | 0.180 | 0.149 | 0.125 | 0.138 | 0.128 | 0.142 | 0.150 | 0.152 | 0.194 |
| 2.249 | 2.187 | 5.47 | 0.005 | 0.024 | 0.082 | 0.159 | 0.217 | 0.235 | 0.213 | 0.162 | 0.129 | 0.115 | 0.122 | 0.113 | 0.123 | 0.104 | 0.108 | 0.123 |
| 4.000 | 3.807 | 9.52 | NaN | 0.024 | 0.083 | 0.159 | 0.218 | 0.233 | 0.208 | 0.161 | 0.125 | 0.107 | 0.102 | 0.099 | 0.094 | 0.084 | 0.104 | 0.092 |
| 7.113 | 6.517 | 16.29 | 0.003 | 0.024 | 0.082 | 0.159 | 0.218 | 0.234 | 0.209 | 0.163 | 0.128 | 0.115 | 0.103 | 0.098 | 0.098 | 0.093 | 0.089 | 0.098 |
| 12.649 | 10.844 | 27.11 | 0.002 | 0.025 | 0.083 | 0.160 | 0.218 | 0.235 | 0.209 | 0.168 | 0.133 | 0.113 | 0.106 | 0.109 | 0.107 | 0.105 | 0.098 | 0.103 |
| 22.494 | 17.205 | 43.01 | 0.003 | 0.024 | 0.082 | 0.159 | 0.218 | 0.235 | 0.211 | 0.164 | 0.131 | 0.110 | 0.107 | 0.099 | 0.103 | 0.105 | 0.096 | 0.098 |
| 40.000 | 25.285 | 63.21 | 0.002 | 0.025 | 0.083 | 0.159 | 0.218 | 0.235 | 0.209 | 0.166 | 0.130 | 0.113 | 0.105 | 0.101 | 0.104 | 0.098 | 0.100 | 0.092 |
| 71.131 | 33.243 | 83.11 | 0.002 | 0.024 | 0.082 | 0.160 | 0.218 | 0.234 | 0.210 | 0.162 | 0.135 | 0.108 | 0.107 | 0.099 | 0.102 | 0.092 | 0.091 | 0.082 |
| 126.491 | 38.307 | 95.77 | 0.002 | 0.024 | 0.083 | 0.159 | 0.218 | 0.235 | 0.209 | 0.162 | 0.130 | 0.110 | 0.110 | 0.102 | 0.100 | 0.097 | 0.104 | 0.102 |
| 224.937 | 39.856 | 99.64 | 0.003 | 0.024 | 0.083 | 0.158 | 0.216 | 0.235 | 0.210 | 0.164 | 0.127 | 0.110 | 0.105 | 0.099 | 0.102 | 0.091 | 0.081 | 0.126 |
| 400.000 | 39.998 | 100.00 | 0.002 | 0.024 | 0.082 | 0.160 | 0.214 | 0.225 | 0.197 | 0.169 | 0.150 | NaN | NaN | 0.133 | 0.111 | NaN | NaN | NaN |

**Table S6** nEUROPt results (only contrast) for SiPM-like IRF simulations.

| **Contrast-to-Noise Ratio** | | | | | | | | | | | | | | | | | | |
| --- | --- | --- | --- | --- | --- | --- | --- | --- | --- | --- | --- | --- | --- | --- | --- | --- | --- | --- |
| **CR [Mcps]** | **CR_sat_ [Mcps]** | **CR_sat_**  **[% Exc. rate]** | **0-0.5 ns** | **0.5-1.0 ns** | **1.0-1.5 ns** | **1.5-2.0 ns** | **2.0-2.5 ns** | **2.5-3.0 ns** | **3.0-3.5 ns** | **3.5-4.0 ns** | **4.0-4.5 ns** | **4.5-5.0 ns** | **5.0-5.5 ns** | **5.5-6.0 ns** | **6.0-6.5 ns** | **6.5-7.0 ns** | **7.0-7.5 ns** | **7.5-8.0 ns** |
|  |  |  | **BEFORE CORRECTION** | | | | | | | | | | | | | | | |
| 0.400 | 0.398 | 1.00 | -0.33 | 7.81 | 23.48 | 33.35 | 27.60 | 15.68 | 6.86 | 4.41 | 1.63 | 0.87 | 1.09 | 0.79 | 0.90 | 0.28 | 0.22 | 0.14 |
| 0.711 | 0.705 | 1.76 | -0.18 | 7.15 | 74.20 | 40.86 | 47.11 | 15.32 | 7.50 | 4.16 | 2.27 | 1.79 | 0.80 | 0.93 | 1.00 | 0.78 | 0.50 | 0.46 |
| 1.265 | 1.245 | 3.11 | 0.47 | 16.02 | 57.11 | 54.65 | 42.12 | 29.24 | 16.46 | 12.32 | 5.68 | 3.94 | 4.17 | 3.19 | 2.57 | 3.06 | 1.99 | 2.72 |
| 2.249 | 2.187 | 5.47 | 1.51 | 18.15 | 62.57 | 73.17 | 58.41 | 51.88 | 34.99 | 21.10 | 13.79 | 5.64 | 6.01 | 3.75 | 3.31 | 2.61 | 2.87 | 2.93 |
| 4.000 | 3.807 | 9.52 | 0.72 | 23.09 | 129.71 | 147.26 | 82.42 | 82.98 | 49.32 | 29.34 | 11.89 | 5.30 | 6.19 | 8.99 | 5.27 | 4.40 | 3.36 | 4.11 |
| 7.113 | 6.517 | 16.29 | 1.41 | 30.72 | 115.56 | 124.46 | 167.62 | 75.17 | 52.86 | 29.71 | 16.34 | 12.59 | 11.46 | 12.44 | 5.30 | 4.90 | 5.46 | 5.15 |
| 12.649 | 10.844 | 27.11 | 1.59 | 49.38 | 216.55 | 226.45 | 200.54 | 66.86 | 37.61 | 36.52 | 16.93 | 13.44 | 12.87 | 14.44 | 11.45 | 7.94 | 9.68 | 6.92 |
| 22.494 | 17.205 | 43.01 | 2.83 | 75.76 | 153.07 | 201.15 | 139.85 | 115.30 | 93.58 | 20.30 | 13.27 | 14.60 | 10.07 | 8.30 | 11.13 | 7.92 | 4.51 | 3.88 |
| 40.000 | 25.285 | 63.21 | 3.28 | 79.49 | 182.77 | 155.32 | 159.76 | 98.42 | 41.72 | 26.50 | 12.16 | 9.25 | 6.35 | 5.27 | 3.29 | 3.10 | 3.01 | 1.60 |
| 71.131 | 33.243 | 83.11 | 3.93 | 200.62 | 331.76 | 140.39 | 169.89 | 126.65 | 107.16 | 56.44 | -0.83 | -10.99 | -40.18 | -7.83 | -6.70 | -18.71 | -31.26 | -17.22 |
| 126.491 | 38.307 | 95.77 | 4.51 | 128.15 | 79.29 | 28.17 | 21.01 | 9.76 | -4.60 | -15.25 | -15.35 | -17.97 | -22.19 | -12.83 | -28.05 | -9.23 | -7.51 | -7.46 |
| 224.937 | 39.856 | 99.64 | 10.37 | 105.87 | -56.22 | -43.29 | -35.74 | -26.59 | -16.18 | -15.33 | -19.47 | -12.38 | -10.90 | -10.43 | -6.53 | -9.07 | -9.02 | -4.37 |
| 400.000 | 39.998 | 100.00 | 14.51 | 28.95 | -98.85 | -53.22 | -15.73 | -5.16 | -2.43 | -1.35 | -0.57 | -0.42 | -0.07 | 0.06 | 0.11 | 0.22 | 0.30 | 0.31 |
|  |  |  | **AFTER CORRECTION** | | | | | | | | | | | | | | | |
| 0.400 | 0.398 | 1.00 | -0.33 | 7.81 | 23.50 | 33.36 | 27.72 | 15.74 | 6.92 | 4.48 | 1.66 | 0.89 | 1.13 | 0.82 | 0.94 | 0.30 | 0.24 | 0.16 |
| 0.711 | 0.705 | 1.76 | -0.18 | 7.15 | 73.94 | 40.98 | 47.51 | 15.45 | 7.60 | 4.24 | 2.33 | 1.84 | 0.85 | 0.98 | 1.04 | 0.83 | 0.54 | 0.49 |
| 1.265 | 1.245 | 3.11 | 0.47 | 16.05 | 57.22 | 54.75 | 42.52 | 29.58 | 16.72 | 12.67 | 5.85 | 4.10 | 4.34 | 3.32 | 2.66 | 3.20 | 2.08 | 2.84 |
| 2.249 | 2.187 | 5.47 | 1.51 | 18.12 | 63.16 | 73.53 | 58.95 | 52.73 | 35.58 | 21.75 | 14.26 | 5.99 | 6.36 | 3.99 | 3.52 | 2.82 | 3.10 | 3.18 |
| 4.000 | 3.807 | 9.52 | 0.72 | 23.09 | 129.28 | 149.72 | 84.84 | 85.11 | 50.10 | 30.66 | 13.03 | 5.92 | 7.05 | 10.70 | 6.27 | 5.51 | 3.84 | 4.96 |
| 7.113 | 6.517 | 16.29 | 1.40 | 30.63 | 116.22 | 130.06 | 175.82 | 79.62 | 57.54 | 32.84 | 18.27 | 15.16 | 14.17 | 15.22 | 6.72 | 6.13 | 7.08 | 6.45 |
| 12.649 | 10.844 | 27.11 | 1.59 | 50.02 | 225.49 | 237.00 | 218.21 | 71.11 | 41.25 | 42.75 | 20.99 | 16.59 | 17.66 | 19.35 | 15.34 | 10.64 | 13.71 | 9.69 |
| 22.494 | 17.205 | 43.01 | 2.83 | 76.93 | 174.17 | 226.86 | 159.72 | 138.03 | 118.56 | 27.41 | 19.29 | 24.93 | 17.81 | 14.66 | 19.73 | 14.90 | 9.55 | 8.14 |
| 40.000 | 25.285 | 63.21 | 3.28 | 76.77 | 205.20 | 224.03 | 216.35 | 133.44 | 64.70 | 53.15 | 31.36 | 36.03 | 25.36 | 21.85 | 16.99 | 18.22 | 16.53 | 14.80 |
| 71.131 | 33.243 | 83.11 | 3.91 | 273.88 | 386.89 | 285.18 | 294.23 | 243.67 | 384.68 | 185.45 | 212.02 | 23.78 | 63.73 | 20.63 | 32.33 | 17.71 | 14.47 | 9.46 |
| 126.491 | 38.307 | 95.77 | 4.51 | 161.99 | 294.79 | 148.40 | 153.24 | 89.03 | 59.44 | 27.37 | 17.97 | 16.77 | 12.78 | 11.24 | 11.14 | 6.72 | 6.17 | 7.66 |
| 224.937 | 39.856 | 99.64 | 10.39 | 135.90 | 130.21 | 84.24 | 58.51 | 34.00 | 16.09 | 11.99 | 11.36 | 4.92 | 4.91 | 4.58 | 3.42 | 3.00 | 3.80 | 3.76 |
| 400.000 | 39.998 | 100.00 | 14.56 | 109.75 | 65.11 | 25.28 | 13.95 | 6.20 | 4.80 | 1.68 | 1.69 | 0.41 | 0.87 | 1.23 | 1.37 | 0.15 | 0.10 | -0.04 |

**Table S7** nEUROPt results (only Contrast-to-Noise Ratio) for SiPM-like IRF simulations.

| **Contrast** | | | | | | | | | | | | | | | | | | |
| --- | --- | --- | --- | --- | --- | --- | --- | --- | --- | --- | --- | --- | --- | --- | --- | --- | --- | --- |
| **CR* [Mcps]** | **CR_sat_* [Mcps]** | **CR_sat_**  **[% Exc. rate]** | **0-0.5 ns** | **0.5-1.0 ns** | **1.0-1.5 ns** | **1.5-2.0 ns** | **2.0-2.5 ns** | **2.5-3.0 ns** | **3.0-3.5 ns** | **3.5-4.0 ns** | **4.0-4.5 ns** | **4.5-5.0 ns** | **5.0-5.5 ns** | **5.5-6.0 ns** | **6.0-6.5 ns** | **6.5-7.0 ns** | **7.0-7.5 ns** | **7.5-8.0 ns** |
|  |  |  | **BEFORE CORRECTION** | | | | | | | | | | | | | | | |
| 1.265 | 1.245 | 3.11 | NaN | 0.012 | 0.044 | 0.107 | 0.164 | 0.185 | 0.158 | 0.132 | 0.087 | 0.093 | 0.078 | 0.064 | 0.081 | 0.084 | 0.091 | 0.072 |
| 40.000 | 25.285 | 63.21 | NaN | NaN | 0.044 | 0.095 | 0.138 | 0.153 | 0.126 | 0.079 | 0.045 | 0.023 | 0.018 | 0.011 | 0.010 | 0.013 | 0.008 | 0.008 |
| 71.131 | 33.243 | 83.11 | NaN | NaN | 0.035 | 0.072 | 0.106 | 0.118 | 0.091 | 0.036 | 0.003 | NaN | NaN | NaN | NaN | NaN | NaN | NaN |
| 126.491 | 38.307 | 95.77 | NaN | NaN | 0.032 | 0.043 | 0.054 | 0.057 | 0.023 | NaN | NaN | NaN | NaN | NaN | NaN | NaN | NaN | NaN |
| 224.937 | 39.856 | 99.64 | NaN | NaN | NaN | NaN | NaN | NaN | NaN | NaN | NaN | NaN | NaN | NaN | NaN | NaN | NaN | NaN |
|  |  |  | **AFTER CORRECTION** | | | | | | | | | | | | | | | |
| 1.265 | 1.245 | 3.11 | NaN | 0.009 | 0.042 | 0.105 | 0.162 | 0.183 | 0.156 | 0.129 | 0.084 | 0.090 | 0.074 | 0.060 | 0.076 | 0.078 | 0.085 | 0.066 |
| 40.000 | 25.285 | 63.21 | NaN | NaN | 0.053 | 0.121 | 0.175 | 0.194 | 0.170 | 0.126 | 0.094 | 0.076 | 0.071 | 0.065 | 0.065 | 0.067 | 0.063 | 0.063 |
| 71.131 | 33.243 | 83.11 | NaN | NaN | 0.049 | 0.117 | 0.172 | 0.191 | 0.166 | 0.119 | 0.092 | 0.071 | 0.069 | 0.066 | 0.060 | 0.062 | 0.063 | 0.062 |
| 126.491 | 38.307 | 95.77 | NaN | NaN | 0.050 | 0.117 | 0.174 | 0.193 | 0.167 | 0.126 | 0.091 | 0.073 | 0.067 | 0.064 | 0.063 | 0.059 | 0.062 | 0.060 |
| 224.937 | 39.856 | 99.64 | NaN | NaN | 0.037 | 0.094 | 0.150 | 0.170 | 0.147 | 0.116 | 0.072 | 0.067 | 0.048 | 0.048 | 0.060 | 0.037 | 0.033 | 0.030 |

**Table S8** nEUROPt results (only contrast) for measurements.

| **Contrast-to-Noise Ratio** | | | | | | | | | | | | | | | | | | |
| --- | --- | --- | --- | --- | --- | --- | --- | --- | --- | --- | --- | --- | --- | --- | --- | --- | --- | --- |
| **CR* [Mcps]** | **CR_sat_* [Mcps]** | **CR_sat_**  **[% Exc. rate]** | **0-0.5 ns** | **0.5-1.0 ns** | **1.0-1.5 ns** | **1.5-2.0 ns** | **2.0-2.5 ns** | **2.5-3.0 ns** | **3.0-3.5 ns** | **3.5-4.0 ns** | **4.0-4.5 ns** | **4.5-5.0 ns** | **5.0-5.5 ns** | **5.5-6.0 ns** | **6.0-6.5 ns** | **6.5-7.0 ns** | **7.0-7.5 ns** | **7.5-8.0 ns** |
|  |  |  | **BEFORE CORRECTION** | | | | | | | | | | | | | | | |
| 1.265 | 1.245 | 3.11 | 0.74 | 1.46 | 10.60 | 12.42 | 19.80 | 18.62 | 13.41 | 8.17 | 3.97 | 3.52 | 3.22 | 2.19 | 2.56 | 3.17 | 4.13 | 1.59 |
| 40.000 | 25.285 | 63.21 | -2.23 | -0.99 | 10.49 | 16.57 | 24.14 | 26.99 | 23.66 | 23.34 | 12.53 | 3.95 | 6.88 | 2.85 | 2.21 | 1.81 | 1.66 | 1.19 |
| 71.131 | 33.243 | 83.11 | -2.13 | -1.86 | 6.71 | 10.86 | 18.68 | 24.81 | 26.98 | 9.91 | 1.27 | -5.17 | -8.57 | -8.93 | -7.49 | -7.22 | -7.18 | -4.90 |
| 126.491 | 38.307 | 95.77 | -2.12 | -1.89 | 3.13 | 3.91 | 5.99 | 7.07 | 3.08 | -2.94 | -9.81 | -10.07 | -9.84 | -9.49 | -8.26 | -8.11 | -8.57 | -7.15 |
| 224.937 | 39.856 | 99.64 | 0.04 | 0.13 | 0.29 | -1.40 | -3.96 | -4.89 | -8.50 | -13.78 | -18.68 | -10.30 | -10.10 | -11.65 | -14.05 | -7.80 | -10.07 | -10.63 |
|  |  |  | **AFTER CORRECTION** | | | | | | | | | | | | | | | |
| 1.265 | 1.245 | 3.11 | 0.67 | 1.13 | 10.50 | 12.35 | 19.78 | 18.38 | 13.18 | 8.14 | 3.84 | 3.40 | 3.08 | 2.06 | 2.44 | 3.00 | 3.96 | 1.48 |
| 40.000 | 25.285 | 63.21 | -2.22 | -0.83 | 22.51 | 27.45 | 34.44 | 37.99 | 38.11 | 48.57 | 25.61 | 14.88 | 30.33 | 19.04 | 11.01 | 9.82 | 13.46 | 10.08 |
| 71.131 | 33.243 | 83.11 | -2.18 | -1.25 | 19.62 | 23.38 | 31.76 | 41.93 | 49.35 | 31.61 | 50.37 | 15.95 | 23.01 | 13.97 | 11.92 | 9.62 | 9.38 | 7.26 |
| 126.491 | 38.307 | 95.77 | -2.06 | -1.12 | 16.59 | 19.66 | 26.53 | 30.57 | 26.29 | 14.08 | 15.51 | 7.74 | 8.45 | 6.68 | 5.52 | 7.39 | 5.33 | 7.26 |
| 224.937 | 39.856 | 99.64 | 0.06 | 0.51 | 13.66 | 16.21 | 17.21 | 18.49 | 11.44 | 9.39 | 5.50 | 2.75 | 2.08 | 3.03 | 4.15 | 1.78 | 1.70 | 2.14 |

**Table S9** nEUROPt results (only Contrast-to-Noise Ratio) for measurements.

* Note that the CR and CR_sat_ here reported are obtained by summing up 10 repetitions of 1 s, thus acquiring curves equivalent to those that could have been obtained at 40 MHz
